# A tiered next-generation risk assessment framework integrating toxicokinetics and NAM-based toxicodynamics: “proof of concept” case study using pyrethroids

**DOI:** 10.1007/s00204-025-04045-9

**Published:** 2025-05-07

**Authors:** Ana Fernandez-Agudo, Jose V. Tarazona

**Affiliations:** 1 Spanish National Environmental Health Center, Instituto de Salud Carlos III, Madrid, Spain; 2 PhD Program in Biomedical Sciences and Public Health, Universidad Nacional de Educación a Distancia (UNED), Madrid, Spain

**Keywords:** Next-generation risk assessment (NGRA), New Alternative Methods (NAMs), Toxicokinetics, Toxicodynamics, Pyrethroids

## Abstract

New Approach Methodologies (NAMs) in Next-Generation Risk Assessment (NGRA), integrating toxicokinetics (TK) with toxicodynamics (TD), provides an accurate evaluation of combined chemical exposures. This study assesses pyrethroids, which pose regulatory challenges due to their widespread use and cumulative exposure risks. A tiered NGRA framework was compared with conventional risk assessment (RA) to evaluate regulatory applicability. In Tier 1, ToxCast data established gene and tissue bioactivity indicators, facilitating hypothesis-driven hazard identification. Tier 2 examined combined risk assessments, rejecting the hypothesis of the same mode of action and highlighting inconsistencies in in vitro data and NOAEL/ADI correlations. Tier 3 applied Margin of Exposure (MoE) analysis and TK modeling to realistic exposure estimations for risk assessment screening based on internal doses, identifying tissue-specific pathways as critical risk drivers. Tier 4 refined bioactivity indicators using TK approaches to improve the NAM-based effect assessment, including an in vitro* vs. *in vivo comparison, with coherent results based on interstitial concentrations, though intracellular estimations remained uncertain. Tier 5 confirmed that dietary exposure in healthy adults is close to but below levels of concern, with bioactivity MoE values remaining below concern thresholds, and in vivo MoEs within the standard safety factors. Nevertheless, the MoEs are insufficient for addressing the additional non-dietary exposure expected from other pyrethroid uses such as biocides or pharmaceuticals. Results demonstrate that NGRA with TK-NAM-based TD offers a nuanced, regulatory-relevant framework for risk assessment. The proposed approach integrates the information on individual pyrethroids using bioactivity indicators; and the re-assessment of regulatory toxicity studies to select organ-relevant NOAELs allowed an improved in vitro*-*in vivo comparison, demonstrating the capacity of bioactivity-based MoEs for combined exposure assessments. This tiered approach provides key insights for regulatory decision-making, establishing a robust model for evaluating pyrethroids and similar chemical classes.

**Supplementary Information:**

The online version contains supplementary material available at 10.1007/s00204-025-04045-9.

## Introduction

The widespread use of pyrethroids, a class of synthetic insecticides, highlights their growing relevance in agricultural and residential applications. Their extensive application has led to increasing exposure through ingestion, inhalation, and dermal contact (Garud et al. 2024). While conventional risk assessment (RA) suggests that individual exposures to pyrethroids are generally below thresholds of concern, combined exposures frequently approach or exceed acceptability limits, raising significant regulatory challenges (Aznar-Alemany and Eljarrat 2020a, b). These concerns are further compounded by their potential neurotoxicity (Soderlund 2020; Andersen et al. 2022a, b) and bioaccumulation in critical tissues such as the brain, liver, and lungs (Aznar-Alemany and Eljarrat 2020a, b; Yanagihara 2023), and the limitations of traditional RA methodologies in addressing cumulative and tissue-specific risks.

This study aims to evaluate the capacity of New Approach Methodologies for Risk Assessment (NGRA) that integrate toxicokinetics (TK) (Elena et al. 2024) with new alternative methods (NAMs) for toxicodynamics (TD) to address these challenges (Cattaneo et al. 2023; Bossier et al. 2020). This case study based on the proposed novel NGRA approach has been set to validate the usefulness of the framework for assessing combined exposures, surpassing the limitations of conventional RA methodologies that rely heavily on acceptable daily intakes (ADIs) and default extrapolation models (Cedergreen et al. 2017; EC, SCOEL 2013). Specifically, this study is designed to offer a detailed exploration of internal dose–response relationships, intracellular bioactivity thresholds, and the cumulative effects of pyrethroid mixtures.

Pyrethroids serve as an ideal case study for the proposed methodology due to their toxicological profile (Ahamad and Kumar 2023), availability of regulatory assessments including toxicological studies and reviews, and information on realistic exposure levels including human biomonitoring (de Alba-Gonzalez et al. 2023). Keeping in mind the need to systematically refine risk evaluations and ensure regulatory applicability (Becker et al. 2007; Gannon et al. 2019), we propose a tiered conceptual framework of increasing complexity for the combined risk assessment of related chemicals based on in vitro bioactivity indicators, which includes options for comparing NAM-based and standard risk assessments (Ji et al. 2024; Ernst et al. 2023). This tiered approach integrates toxicokinetic modeling and bioactivity data to highlight key mechanistic pathways and assess the combined risks posed by the combined exposure to related chemicals.

The key elements of the proposed tiered NGRA approach that we want to try out in this manuscript include its ability to compare and refine risk assessments based on short-term bioactivity thresholds with chronic toxicity thresholds, to refine exposure levels using available TK tools, and to assess both the risk of each active substance and that of combined exposures (Bhateria et al. 2024; Figueiredo & Bernardete Ferraz Spisso 2024). The proposed approach aims to compare NGRA and conventional assessments (Bearth et al. 2025) through the integration of available toxicokinetic tools and models to estimate the internal concentrations achieved during the in vivo and in vitro testing and those expected at realistic exposure conditions at a population level. The case study with pyrethroids explores the capacity of this tiered framework to offer a scalable and transferable model for evaluating chemical classes with complex exposure profiles.

By highlighting the capacity of NGRA to enhance regulatory standards and public health protections, this study would contribute to the evolving landscape of chemical safety evaluation. The methodological advancements presented here not only refine pyrethroid risk assessments but also would establish a blueprint for implementing NGRA for combined exposure assessment across diverse regulatory contexts and chemical classes.

## Materials and methods

This study employed an integrated approach to assess the bioactivity and toxicokinetic profiles of multiple pyrethroids. The evaluation encompassed in vivo and in vitro data, using ToxCast bioactivity assays and TK modeling to estimate internal concentrations in the toxicity studies and at realistic exposure levels.

The comparison of estimated exposure levels based on human biomonitoring and food monitoring (de Alba_Gonzalez et al. 2023) indicated that dietary exposure estimations for six pyrethroids (bifenthrin, cyfluthrin, cypermethrin, deltamethrin, lambda-cyhalothrin, and permethrin) based on EFSA`s middle bound PRIMo approach represent a good estimation of actual overall pyrethroid exposure levels for EU adults. Therefore, these exposure estimations were used for this assessment.

**Fig. 1:**
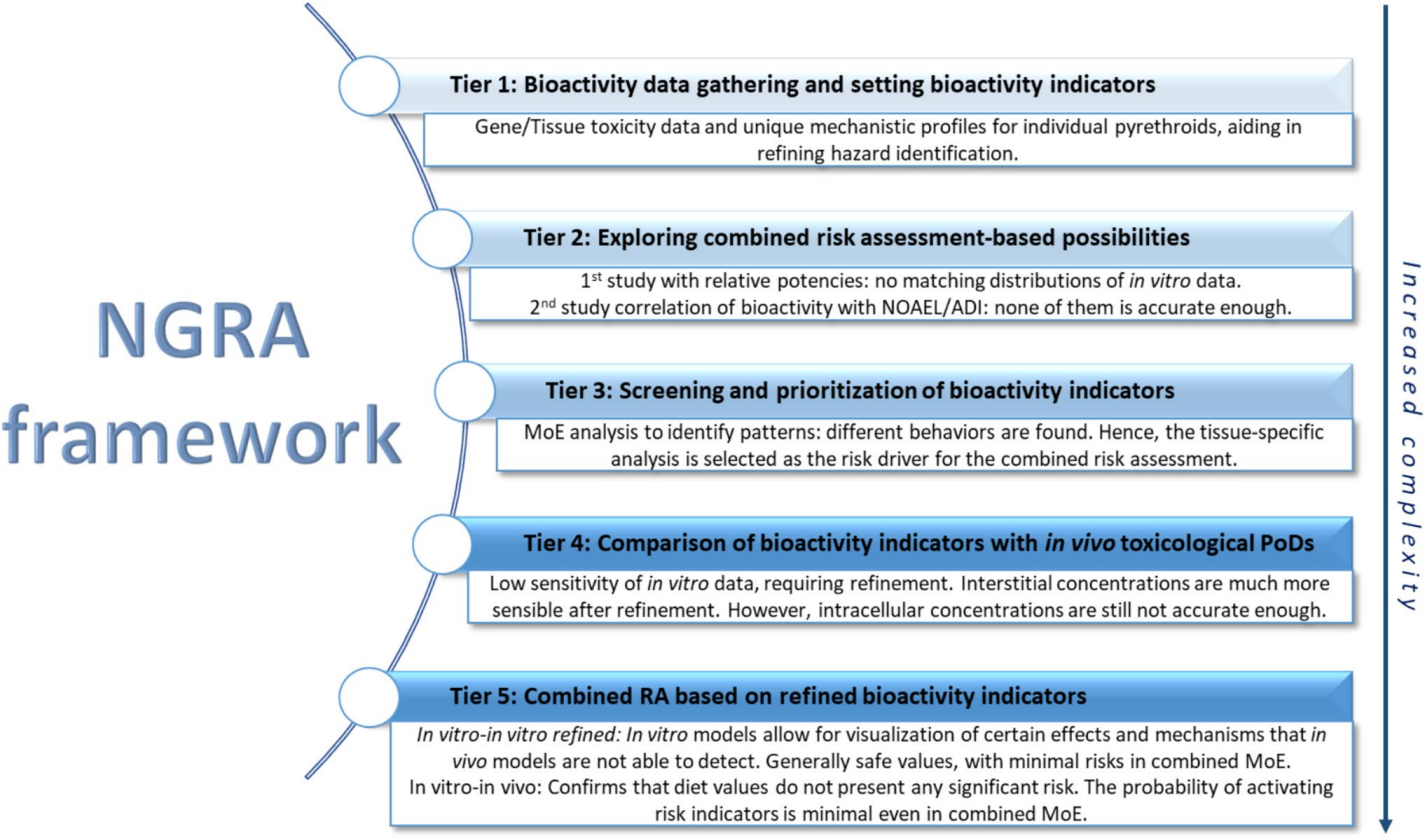
Next-generation risk assessment framework—proof-of-concept case study design. Graphical abstract of methods and tiers used for this novel NGRA increasing complexity

Figure 1 summarizes the conceptual framework developed for this assessment, as a tiered approach with different comparisons and refinements of increased complexity:

### Tier 1: bioactivity data gathering and setting bioactivity indicators

Bioactivity data for the six pyrethroids (bifenthrin, cyfluthrin, cypermethrin, deltamethrin, l-cyhalothrin, and permethrin) were obtained from the ToxCast database (CompTox Chemicals Dashboard 2024). ToxCast provides assay-based measures of bioactivity for numerous chemicals across different biological pathways. For each pyrethroid, bioactivity assay data were categorized by key tissue and gene categories following the ToxCast descriptors.

For tissue-specific analyses, assays were grouped by their relevance to various tissue systems such as breast, kidney, liver, lung, immune system, brain, ovary, pancreas, prostate, skin, and vascular systems. Similarly, for gene-specific analyses, assays were categorized based on their involvement in key biological functions such as androgen receptor signaling, apoptosis, cytochrome P450 activity, DNA damage, neuroreceptors, and thyroid hormone receptors, among others. Average AC_50_ values were calculated within each category and used as indicators to assess the bioactivity patterns of the pyrethroids.

### Tier 2: exploring combined risk assessment-based possibilities

Relative potencies for each pyrethroid were calculated for exploring the hypothesis of the same mode of action.

For the ToxCast data, the averaged AC_50_ values were normalized against the pyrethroid with the highest toxicity in each gene or tissue category. A relative potency value of 1 was assigned to the most potent pyrethroid in each category, with other values scaled accordingly. These calculations were performed using the following equation:

1 $$relative\;potency = \frac{{most\;potent\;value}}{{organ/pathway\;specific\;value}}$$

Radial charts were created to visualize and compare the bioactivity patterns of the pyrethroids. Relative potencies were plotted emphasizing that higher AC_50_ values indicate lower toxicity. Pyrethroids without activity in a specific gene or tissue were assigned the highest AC_50_ value, represented as zero potency on the charts.

To draw comparisons with the bioactivity-based relative potencies derived from ToxCast data, ADI and NOAEL values for each pyrethroid were collected (Table 1) from the European Chemicals Agency (ECHA) and the European Food Safety Authority (EFSA) reports: bifenthrin (EFSA 2011), cyfluthrin (ECHA 2018), cypermethrin (ECHA 2017), deltamethrin (ECHA 2011), l-cyhalothrin (EFSA 2014), and permethrin (ECHA 2014). The ADIs and NOAELs relative potencies were calculated using Eq. 1. Correlations between ToxCast bioactivity data and ADI values were evaluated by plotting relative potencies derived from ToxCast data against those calculated using ADIs. Similar analyses were conducted to compare relative potencies derived from ToxCast data with NOAEL values for specific tissues/organs.

**Table 1:** Selected ADI and NOAEL values for each pyrethroid across various tissues: including the brain, kidney, liver, lung, and skin. Input data for the different evaluations

NOAEL (MG/KG BW/D)							ADI (MG/KG BW/D)
Substance	Peripheral – general	Brain – neuro repeated	Kidney – long term	Liver – long term	Lung – long term	Skin – short term	General
Bifenthrin (EFSA 2011)	1.5	2.9	4.7	4.7	4.7	1.5	0.015
Cyfluthrin (ECHA 2018)	2	2	12	12	12	6.5	0.02
Cypermethrin (ECHA 2017)	5	20	5	5	5	12.5	0.05
Deltamethrin (ECHA 2011)	1	4	1	1	1	–	0.36
L-cyhalothrin (EFSA 2014)	0.25	0.5	1.7	1.7	1.7	0.5	0.0025
Permethrin (ECHA 2014)	5	–	5	5	5	–	0.05

### Tier 3: screening and prioritization of bioactivity indicators

#### Plasma concentrations simulation for realistic dietary exposure levels

A previous study (de Alba-Gonzalez et al. 2023) comparing realistic prospective exposure estimations with human biomonitoring was used for selecting realistic dietary exposure levels. Based on the study results the maximum concentration of the middle-bound doses from the exposure data estimated from the annual EFSA reports were selected to represent the maximum realistic exposure levels in European adults (de Alba-Gonzalez et al. 2023). The data extracted was from the years 2016, 2017, 2019, 2020 (bifenthrin), 2016, 2017, 2018, 2019, 2020 (cyfluthrin, cypermethrin, deltamethrin, l-cyhalothrin, and permethrin).

PKSim, a physiologically based pharmacokinetic (PBPK) modeling software, was employed to simulate plasma concentrations (Willmann et al. 2003) for each pyrethroid and the interstitial and intracellular levels for each tissue of interest. Parameters for human physiology, including age, weight, gender, and body mass index, were selected from the literature. The simulated population consisted of 139 males - European, 30 years old, 73 kg - (Quindroit et al. 2019). Pharmacokinetic parameters and data on absorption, distribution, metabolism, and excretion (ADME) were sourced from prior research (Knaak et al. 2012; DrugBank 2024; Sethi et al. 2015; de Alba-Gonzalez et al. 2023). Each pyrethroid was defined in the model according to its physicochemical properties, ADME characteristics, and dosing regimens. Simulations were set up for an oral dosing scenario involving a single dose every 24 h over a period of three months. The specific parameters and dosing regimen are presented in Table 2. The outputs of interest included C_max_, C_min_, and the area under the concentration–time curve (AUC), with a focus on the maximum concentration (C_max_) as the exposure metric of interest.

**Table 2:** Human parameters for PKSim. Physiological (e.g., gender, weight, height, age), ADME parameters and dosing scenarios for each pyrethroid are presented. * –: no available information for these variables

PKSIM parameters	Bifenthrin	Cyfluthrin	Cypermethrin	Deltamethrin	L-cyhalothrin	Permethrin
Molecular weight (g/mol) (DrugBank 2024)	422.9	434.3	416.3	505.2	449.85	391.3
Water solubility (mg/mL) (DrugBank 2024)	4.4E-05	6.57E-04	8.64E-04	1.3E-06	5E-03	6.91E-05
Fraction unbound to albumin (%) (Sethi et al. 2015)	10	10	10	10	10	10
Log P (DrugBank 2024)	5.71	5.93	5.81	5.74	5.5	6.24
pKa (DrugBank 2024)	−7.1	10.49	10.65	10.65	–	−3.7
Intestinal perm (cm/s)	4.37E-4	1.04E-4	1.09E-4	1.64E-4	1.28E-4	4.4E-4
Km (uM) (Knaak et al. 2012)	7.83	37.20	41.3	41.5	30.6	27.7
Kcat (1/h) (DrugBank 2024)	–	15	9	11	60	9.1
Exposure concentration (mg/kg bw/d) (de Alba-Gonzalez et al. 2023)	1.35E-04	1.12E-04	1.14E-03	2E-04	2.42E-04	8.58E-04

The simulated plasma concentrations were validated against observed experimental data. This involved reverse estimation to compare model-predicted urine concentrations to observed exposure data. These estimations were performed via a molar correction of the pyrethroid Deltamethrin concentration to extract its value for its metabolites. The obtained concentration was then compared against the urine estimations from de Alba-Gonzalez et al. 2023.

#### Screening and prioritization of pyrethroid’s risk based on in vitro bioactivity

The margin of exposure (MoE) approach was used as a risk indicator. MoEs were calculated by dividing the bioactivity indicators based on the AC_50_ values from ToxCast by the exposure levels represented by the C_max_ values derived from the PBPK simulations. MoEs were grouped as ranges for this screening assessment: MoE < 100 posing higher concern, 100 < MoE < 1000, 1000 < MoE < 100,000, and MoE > 100,000 representing the lowest concern.

Further investigation into the mechanisms of action for pyrethroids with MoE values below 100 was conducted through data mining of the ToxCast results. Specifically, the study examined pathways and genes related to functional neural network activity, spontaneous neural activity, and gene expression regulation. A detailed analysis was performed to determine whether other pyrethroids exhibited similar bioactivity profiles but with varying degrees of potency (AOP-wiki n.d., Karaca et al. 2020; Lago & Puzzi 2019; Smith et al. 1997; Zhou et al. 2021; Zhang et al. 2022; Coussens et al. 2000; Corley et al. 2018).

### Tier 4: comparison of bioactivity indicators with in vivo toxicological points of departure (PoD)

To compare in vitro and in vivo toxicological PoD, additional PBPK simulations were performed using as exposure level the NOAEL values for each tissue and pyrethroid. The estimated C_max_ values at the NOAEL for various tissues were used to generate two comparative graphs; plotting bioactivity indicators based on the AC_50_ values against interstitial concentrations and against intracellular concentrations. The equipotency line was plotted, with 10-fold and 100-fold deviation lines to facilitate interpretation of the data.

#### Refinement of bioactivity indicators

To refine the relationship between in vitro and in vivo PoDs, Tox21 GeneBLAzer bioassays (Fischer et al. 2017; Ulrich et al. 2017) was used to model freely dissolved (C_free_), cellular (C_cell_), and membrane-bound (C_mem_) concentrations. The bioactivity indicators based on the AC_50_ values for each target tissue were used as the nominal concentrations (C_nom_) for the modelling. The log DBSA/w and log Dlip/w [L/L], pH 7.4, 37 °C values of the compounds Cyfluthrin, Deltamethrin, and L-Cyhalothrin were extrapolated from the partition coefficients due to a lack of LSER predictions for these substances.

The comparison of in vitro and in vivo PoDs was refined by replacing the bioactivity indicators based on nominal concentrations by the estimated free and intracellular levels.

### Tier 5: combined RA based on refined bioactivity indicators

MoEs for realistic pyrethroid exposure levels were calculated by dividing the refined bioactivity indicators based on free and intracellular concentrations by the C_max_ values for realistic pyrethroid exposure levels estimated at Tier 3. The MoEs were grouped in the following ranges MoE < 1 indicating high probability of bioactivation, 1 < MoE < 10, 10 < MoE < 100, and MoE > 100 representing progressive assessment factors.

The combined MoE was calculated using Eq. 2 to estimate the likelihood of bioactivation in the case of the combined effects of the six pyrethroids as a mixture.

2 $$combined\;MOE= \frac{1}{1/\Sigma (1/MoE)}$$

To compare the risk characterization based on refined bioactivity with the risk levels estimated from in vivo animal studies, a second set of MoEs was calculated using as PoD the C_max_ values estimated for exposures at the NOAEL derived from the PBPK simulations in Tier 4, obtaining an in vivo*-*in vivo estimation. The MoEs were grouped in the following ranges MoE < 1 indicating exposures above the PoD, 1 < MoE < 10, 10 < MoE < 100, and MoE > 100 representing progressive assessment factors.

Lastly, a combined MoE was calculated using Eq. 2 to estimate the probability of bioactivation in the case of the combined effects linked to dietary exposure to the six pyrethroids.

## Results

The study combines toxicokinetics (TKplate) and toxicodynamics (ToxCast bioactivity evaluation) approaches to assess the combined risk of pyrethroids exposure, proposing a tiered framework that also facilitates the comparison of NAM-based NGRA with risk assessments based on animal studies.

### Tier 1: bioactivity data gathering and setting bioactivity indicators

Bioactivity data for six pyrethroids (bifenthrin, cyfluthrin, cypermethrin, deltamethrin, l-cyhalothrin, and permethrin) were obtained from the ToxCast database. For each pyrethroid, bioactivity assay data were categorized by gene category and tissue category: bifenthrin (Appendix 1, Tables S1 And S2), cyfluthrin (Appendix 1, tables S3 and S4), cypermethrin (Appendix 1, tables S5 and S6), deltamethrin (Appendix 1, tables S7 and S8), l-cyhalothrin (Appendix 1, tables S9 and S10), and permethrin (Appendix 1, tables S11 and S12).

Average AC_50_ values were calculated for each category and each pyrethroid to be used as bioactivity indicators for identifying the toxicity patterns of the pyrethroids and selecting PoDs for risk assessment. Data filtering included the exclusion of assays with insufficient or inconsistent data to ensure the reliability of average AC_50_ values (Tables 3 and 4).

**Table 3:** Average AC_50_ values per tissue category. Average AC_50_ values extracted from the ToxCast database of each of the pyrethroids of interest (bifenthrin, cyfluthrin, cypermethrin, deltamethrin, l-cyhalothrin, permethrin) and classified by tailor-made tissue categories. The cells in the blank indicate that the AC_50_ value was not available for those tissues

Average AC_50_ per tissue category (uM)	Bifenthrin	Cyfluthrin	Cypermethrin	Deltamethrin	L-cyhalothrin	Permethrin
Breast	52.24	75.14	51.02	69.69	40.00	76.02
Intestine	26.84		19.46	59.82		46.82
Kidney	30.09	33.44	33.56	27.49	35.90	33.62
Liver	29.75	22.82	26.03	15.57	13.19	26.84
Lung				0.96	0.59	
Immune system	14.70	41.30	66.95	60.38	44.09	
Brain	3.12	4.60	8.21	11.03	7.60	21.14
Ovary	52.61	32.06	9.96	52.55	53.26	3.36
Pancreas			20.44			
Prostate function	39.40	38.91	51.28	43.66		29.41
Skin	5.78	12.42		2.59	1.55	5.78
Vascular system	7.14	12.23	8.43	8.48	8.25	7.14

**Table 4:** Average AC_50_ values per tissue category. Average AC_50_ values extracted from ToxCast database of each of the pyrethroids of interest (bifenthrin, cyfluthrin, cypermethrin, deltamethrin, l-cyhalothrin, permethrin) and classified by tailor-made gene categories. The cells in the blank indicate that the AC_50_ value was not available for those tissues

Average AC_50_ per gene category (uM)	Bifenthrin	Cyfluthrin	Cypermethrin	Deltamethrin	L-cyhalothrin	Permethrin
Androgen receptor	22.54	17.74	18.92	23.60	6.96	38.27
Apoptosis			50.00	0.32	0.35	
ATP binding	36.85	32.02	30.34			
Collagen		0.10		4.86		5.08
Cytochrome	18.39	19.14	12.58	1.09	0.91	14.75
DNA	18.39	21.71	39.46	49.43	44.04	
Epidermal growth factor		50.00	50.00			
Estrogen	48.33	28.63	43.91	50.00	44.97	34.81
Fatty acid	34.10				2.98	
Glucuronidation	16.10	10.00	10.03			9.20
Heat shock protein		17.23		67.64	51.13	
Immune system receptor	8.58	6.69	7.08	6.59	6.86	4.47
Inflammation	7.11	16.33	0.52	14.05	13.16	6.41
Insulin	34.39		50.00			
Artherosclerosis	3.85			3.97		
Leukemia				1.99	4.56	
Liver		26.08				
Thrombosis	10.02	32.19		6.37	4.60	8.25
Neuroreceptor	0.33	3.66	12.98	17.67		73.41
Nuclear receptor	12.08	12.89	11.39	13.55	7.04	14.21
Plasminogen			10.43	4.43	0.62	
Progesterone receptor	20.35	39.24	21.24	15.21	26.98	10.65
Proliferation	5.12			28.75	2.98	20.00
Prostaglandin	4.12	4.55	4.42	2.14	0.69	3.87
Protein coding	9.23					
Protein kinase		26.55	50.00			6.34
Protein phosphatase			3.45			
Proteolysis	3.32		11.64		1.93	4.05
Sulfotransferase	5.45	50.00				
Thyroid hormone receptor	17.73	38.03	47.33	74.77	35.75	100.00

### Tier 2: exploring combined risk assessment-based possibilities

Relative potencies of the averaged AC_50_ values of each pyrethroid *vs.* the pyrethroid with the highest toxicity within each category are presented in Tables 5 and 6.

**Table 5:** AC_50_ Relative potencies of pyrethroids. Tissue categorization. The ToxCast data were extracted for each pyrethroid bioactivity summary

AC_50_ relative potencies	Bifenthrin	Cyfluthrin	Cypermethrin	Deltamethrin	L-cyhalothrin	Permethrin
Breast	0.77	0.53	0.78	0.57	1.00	0.53
Intestine	0.73		1.00	0.33		0.42
Kidney	0.91	0.82	0.82	1.00	0.77	0.82
Liver	0.44	0.58	0.51	0.85	1.00	0.49
Lung				0.61	1.00	
Immune system	1.00	0.36	0.22	0.24	0.33	
Brain	1.00	0.68	0.38	0.28	0.41	0.15
Ovary	0.06	0.10	0.34	0.06	0.06	1.00
Pancreas			1.00			
Prostate function	0.75	0.76	0.57	0.67		1.00
Skin	0.27	0.12		0.60	1.00	0.27
Vascular system	1.00	0.58	0.85	0.84	0.87	1.00

**Table 6:** AC_50_ Relative potencies of pyrethroids. Gene categorization. The ToxCast data was extracted for each pyrethroid bioactivity summary

AC_50_ relative potencies	Bifenthrin	Cyfluthrin	Cypermethrin	Deltamethrin	L-cyhalothrin	Permethrin
Androgen receptor	0.31	0.39	0.37	0.29	1.00	0.18
Apoptosis			0.01	1.00	0.91	
ATP binding	0.82	0.95	1.00			
Collagen		1.00		0.02		0.02
Cytochrome	0.05	0.05	0.07	0.83	1.00	0.06
DNA	1.00	0.85	0.47	0.37	0.42	
Epidermal growth factor		1.00	1.00			
Estrogen	0.59	1.00	0.65	0.57	0.64	0.82
Fatty acid	0.09				1.00	
Glucuronidation	0.57	0.92	0.92			1.00
Heat shock protein		1.00		0.25	0.34	
Immune system receptor	0.52	0.67	0.63	0.68	0.65	1.00
Inflammation	0.07	0.03	1.00	0.04	0.04	0.08
Insulin	1.00		0.69			
Artherosclerosis	1.00			0.97		
Leukemia				1.00	0.44	
Liver		1.00				
Thrombosis	0.46	0.14		0.72	1.00	0.56
Neuroreceptor	1.00	0.09	0.03	0.02		
Nuclear receptor	0.58	0.55	0.62	0.52	1.00	0.50
Plasminogen			0.06	0.14	1.00	
Progesterone receptor	0.52	0.27	0.50	0.70	0.39	1.00
Proliferation	0.58			0.10	1.00	0.15
Prostaglandin	0.17	0.15	0.16	0.32	1.00	0.18
Protein coding	1.00					
Protein kinase		0.24	0.13			1.00
Protein phosphatase			1.00			
Proteolysis	0.58		0.17		1.00	0.48
Sulfotransferase	1.00	0.11				
Thyroid hormone receptor	1.00	0.47	0.37	0.24	0.50	0.18

**Fig. 2:**
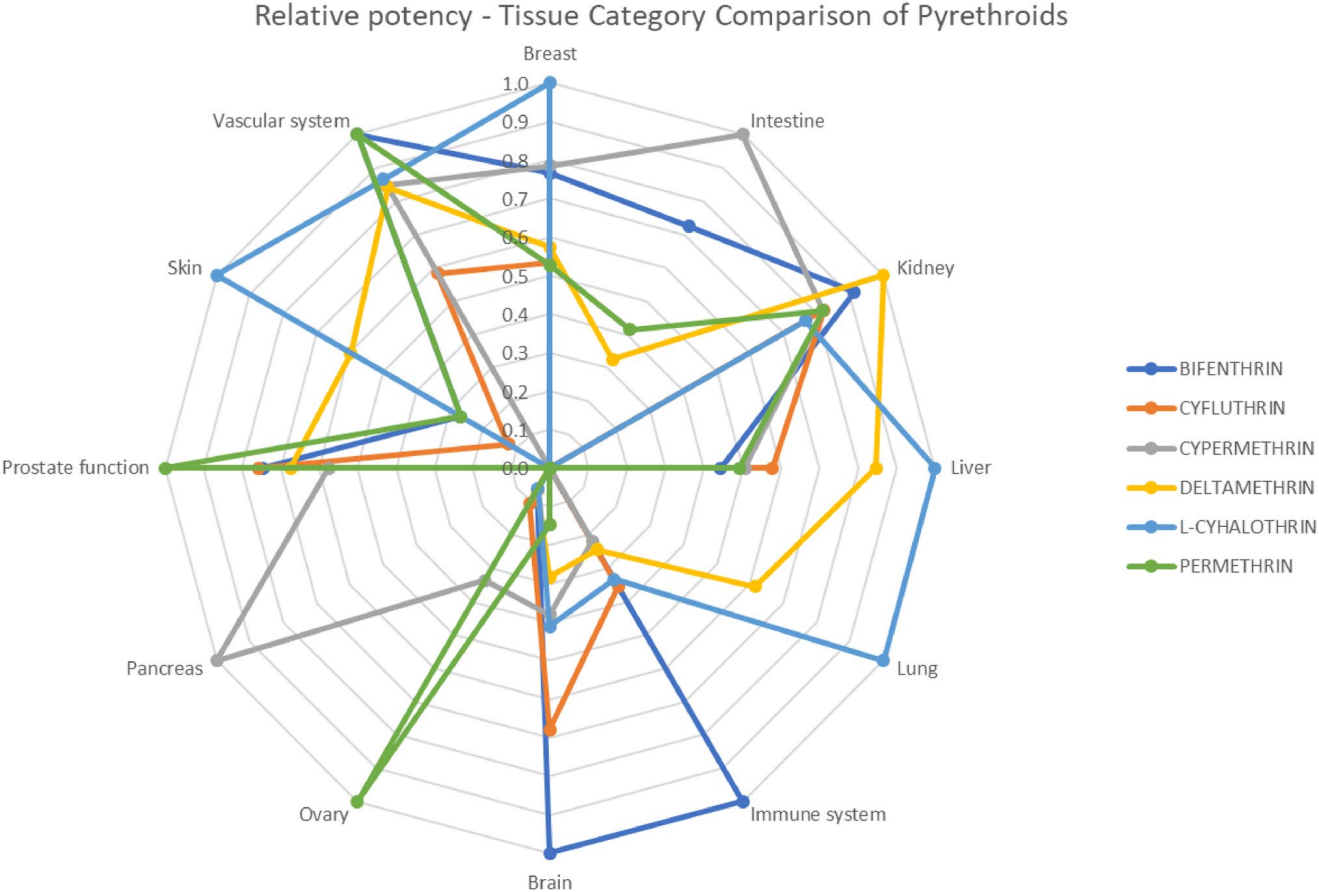
Radial chart of pyrethroid bioactivity by relative potency of the average AC_50_. Tissue category comparison

**Fig. 3:**
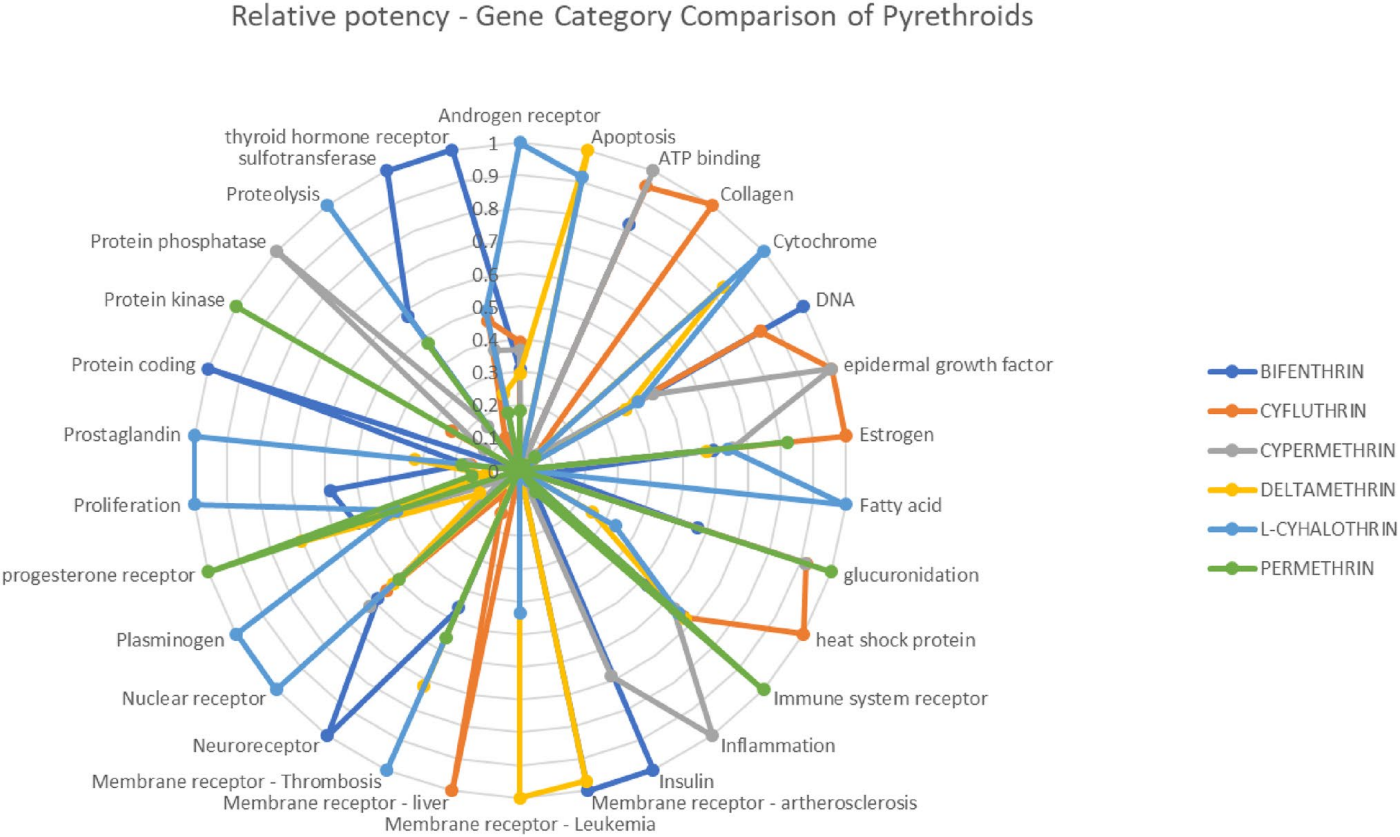
Radial chart of pyrethroid bioactivity by relative potency of the average AC_50_. Gene category comparison

A radial chart was generated from this data to compare the activities of the pyrethroids using these average AC_50_ values (Figs. 2 and 3) in the form of relative potencies. Only studies that showed activity were included in this analysis.

The results of these bioactivity screenings reveal different pathways and relative potencies depending on the affected gene pathways and tissue-specific routes.

**Fig. 4:**
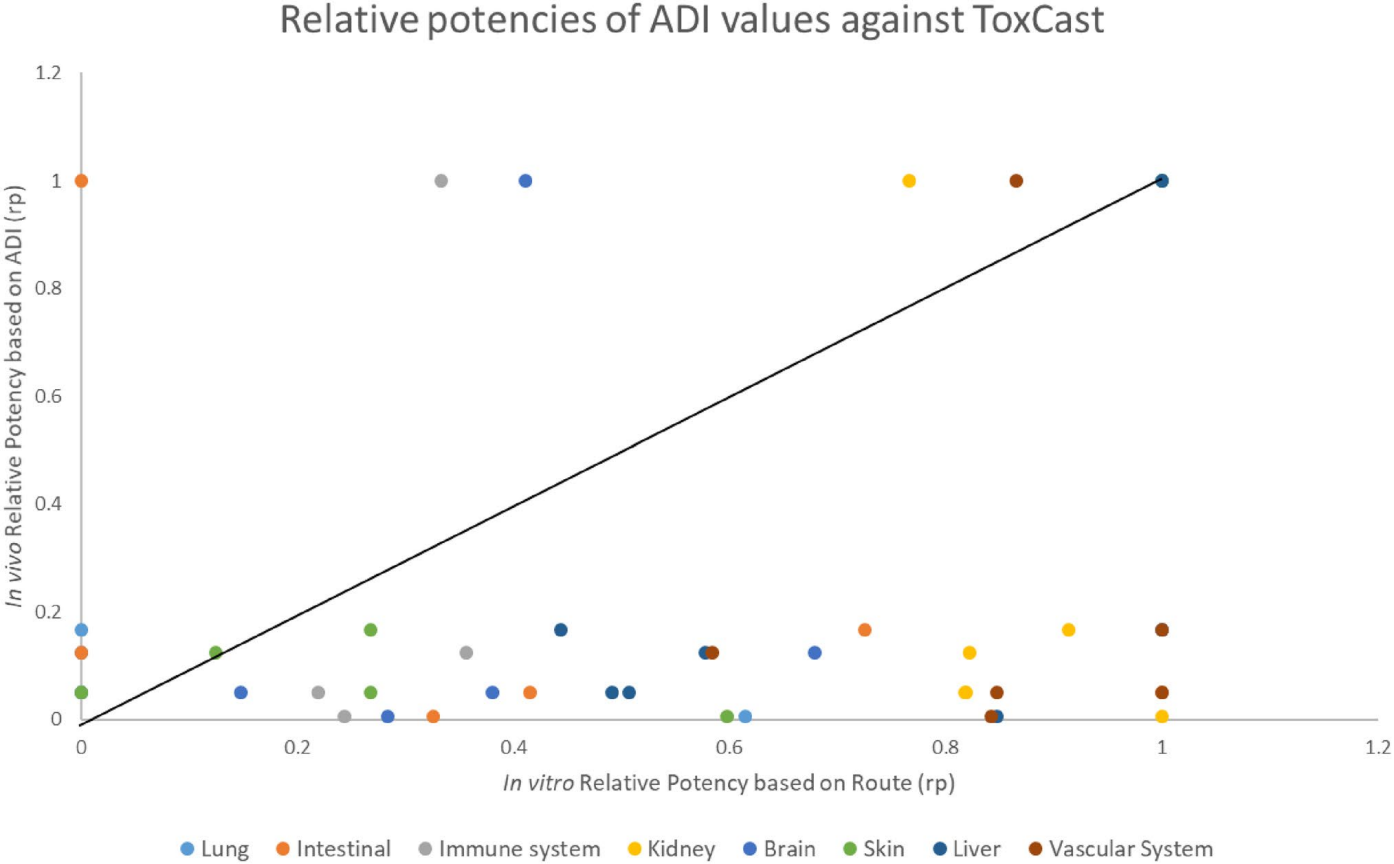
Correlation of ToxCast relative potencies with Acceptable Daily Intakes (ADI) relative potencies. Correlation between relative potencies (ratio vs. the most toxic substance for the endpoint) for apical effects and ToxCast bioactivities. The black line indicates equipotency

**Fig. 5:**
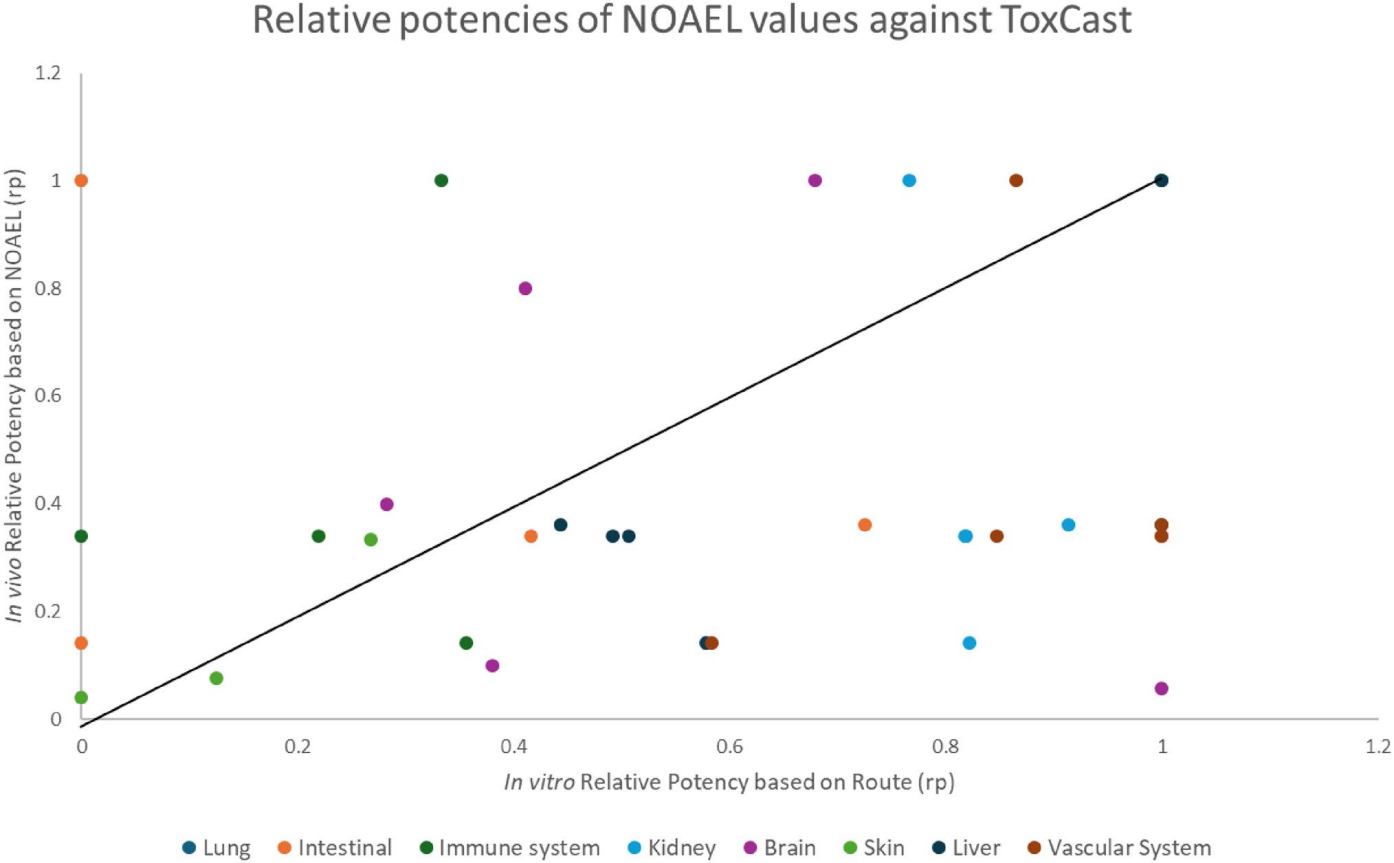
Correlation of ToxCast relative potencies with NOAEL relative potencies. Correlation between relative potencies (ratio vs. the most toxic substance for the endpoint) for apical effects and ToxCast bioactivities. The black line indicates equipotency

Bioactivity relative potencies were compared with in vivo relative potencies, using the regulatory guidance value, ADI, (Fig. 4) and the NOAELs as PoDs for different tissues/organs extracted from the regulatory assessment (Fig. 5).

Data revealed a more homogenic distribution around the equipotency line in the case of the NOAEL values for specific tissues/organs (Fig. 5) but lacking a clear relationship. Therefore, the hypothesis of the same mode of action was rejected, and the option for a combined risk assessment based on relative potencies was dismissed.

In line with the proposed framework, the assessment progressed to the next tier, with detailed mechanistic assessments; and the tissue/organ NOAELs were used for further in vitro to in vivo comparisons.

### Tier 3: screening and prioritization of bioactivity indicators

#### Plasma concentrations simulation for realistic dietary exposure levels

Validation results indicated consistency between predicted and observed concentrations, suggesting that no adjustments to the model were necessary. Such validation consisted of obtaining the urine levels that belong to the selected maximum concentration from the middle bound of 0.0002 mg/kg bw/d and translating it to its metabolites, obtaining 2.65 ug/L for the model-predicted urine concentrations. These predicted concentrations were then compared to the observed exposure data from Alba-Gonzalez et al. 2023. The 2.65 ug/L was between the 75th and the 90th percentile for the population with higher measured levels for Cis DBCA data in urine, corresponding to a range between 1.25 – 3.34 ug/L. These results suggest that the model predictions are accurate and can be considered to be a realistic scenario for dietary exposure since it corresponds to the intermediate value between the 75th and 90th percentiles of the most exposed population.

#### Screening and prioritization of pyrethroid’s risk based on in vitro bioactivity

Realistic internal exposure values represented the C_max_ for plasmatic, interstitial and intracellular levels in different organs, were estimated by PKSim simulations and compared with the bioactivity indicators used as in vitro PoDs. The MoE approach was used for the risk characterization screening, which was supported by color codes and then calculated by dividing the bioactivity indicators based on AC_50_ values from ToxCast by the C_max_ values obtained from PKSim simulations. The analysis was conducted across the multiple tissues of interest. These results are summarized in Table 7. All compounds showed MoE values above 10.000 in the interstitial compartment of each of the selected organs, suggesting low concern. However, intracellular concentrations for several organs showed MoE values under 100, requiring further assessment.

**Table 7:**
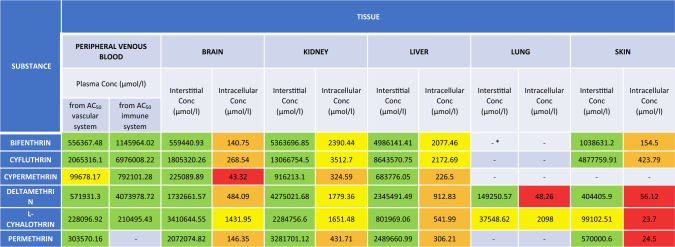
Margin of Exposure (MoE) for tissue-specific bioactivity. RA characterization for bifenthrin, cyfluthrin, cypermethrin, deltamethrin, l-cyhalothrin, permethrin at realistic exposure levels (de Alba-Gonzalez et al., 2023). Each cell represents the MoE (range between the tissue toxicity endpoint and the internal exposure predicted by the TK model) of a specific compound in a particular tissue. The color of each cell indicates the risk level according to the following code: Red (100000)

*Exposure range not calculated due to the lack of AC_50_ bioactivity values.

Further analysis was conducted on compounds with a MoE below 100. The primary goal was to investigate the underlying mechanisms impacting tissues and genes where these compounds showed significant activity. This involved an in-depth review of bioactivity data across various gene and pathway categories to identify potential patterns. The main results are described in the Suppl. Materials, Appendix 2. After identifying these potential common patterns, a graphical analysis was generated (Table 8) to examine the data further, with a focus on exploring the potential consequences and adverse effects arising from the activation of these pathways and genes:

**Table 8:** Consequences and adverse effects from the target tissues with a Margin of Exposure (MoE) under 100 from exposure data. This information was mainly extracted from AOP-wiki (AOP-wiki, n.d.). When no information was available, a bibliographic search was performed

Pyrethroids under 100 moe from exposure data			
Substance	Target tissue	Consequences	Adverse effects
*Cypermethrin*	Brain	Activation of extracellular functional neural network activity and spontaneous neural activity	Functional Neural Network Activity: Excitotoxicity and Neuronal Death: Binding of agonists to ionotropic glutamate receptors causes excitotoxicity, leading to neuronal cell death and impairments in learning and memory. Enzyme Inhibition and Activation: Inhibition of AChE and activation of CYP2E1 result in sensory axonal neuropathy and increased mortality. Gene-Induced Effects: Genes like *elavl3*, *sox10*, and *mbp* influence various neuronal outcomes Spontaneous Neural Activity: NMDAR Antagonism and Development: Antagonism of NMDARs during brain development impairs learning and memory. Nicotinic Receptor Activation: Causes abnormal role changes in bees, leading to colony failure. Chemical Binding and Receptor Antagonism: Binding to ionotropic glutamate receptors and inhibition of Na + /I- symporter impairs learning and memory and binding to thiol/selenoproteins increases oxidative stress susceptibility, affecting cognition. Metabolic Disruptions: Cholesterol/glucose dysmetabolism drives Tau-related pathways linked to Alzheimer’s, while iron accumulation leads to neurological disorders. Hormonal Receptor Antagonism: Estrogen and retinoic acid receptor antagonism increase risks of autism-like behavior and cognitive impairment
Deltamethrin	Lung	Regulation of gene expression (MHC class II)	MHC Class II: Activates proteins, dendritic cells, and T-cells, leading to increased allergic respiratory hypersensitivity due to covalent protein binding
Deltamethrin	Skin	Regulation of gene expression (COL3A1 and TIMP2)	COL3A1: Cushing syndrome is associated with increased expression levels of skin COL1A2, COL2A1, COL3A1 mRNAs (which are correlated with increased expression level of skin GH mRNA). Mutations in the COL3A1 gene result in Ehlers-Danlos syndrome type IV and alterations in the size and distribution of the major collagen fibrils of the dermis. Its effect on aging in primary human dermal fibroblasts is under study TIMP: The imbalance of MMP-2/TIMP-2 and MMP-9/TIMP-1 contributes to the skin disorder of collagen deposition in diabetes patients, providing ideas for managing diabetes skin complications early. Its effect on aging in primary human dermal fibroblasts is under study
*L-*cyhalothrin	Skin	Regulation of gene expression (TIMP2, PLAU, MMP1, MMP9) and cell proliferation	MMP1: facilitates cell growth and migration in vitro by phosphorylating the AKT pathway and promotes tumor progression in head and neck squamous cell carcinoma. Its effect on aging in primary human dermal fibroblasts is under study PLAU: PLAUR initiates proteolytic cascade that converts plasminogen to plasmin, playing a key role in the regulation of cell migration, proliferation and adhesion processes in cancer and inflammation MMP9: hyperproliferation of oncogene-expressing keratinocytes, enhances malignant expression of dysplasia into Frank carcinomas, and affects differentiation/characteristics of emerging tumors. The imbalance of MMP-2/TIMP-2 and MMP-9/TIMP-1 contributes to the skin disorder of collagen deposition in diabetes patients, providing ideas for managing diabetes skin complications early. Its effect on aging in primary human dermal fibroblasts is under study
Permethrin	Skin	Regulation of gene expression (COL3A1, EGFR) and cell proliferation	COL3A1: refer to effects in Deltamethrin

The results confirm both, unique and shared biological pathways, targeted by these compounds; with certain pyrethroids exhibiting highly tissue-specific effects while other pathways are related to mechanisms of broader systemic concern. Considering the variability among compounds in their bioactivity patterns, a tissue-specific analysis is selected as the risk driver for the combined risk assessment in the following tiers.

### Tier 4: comparison of bioactivity indicators with in vivo toxicological points of departure (PoD)

**Fig. 6:**
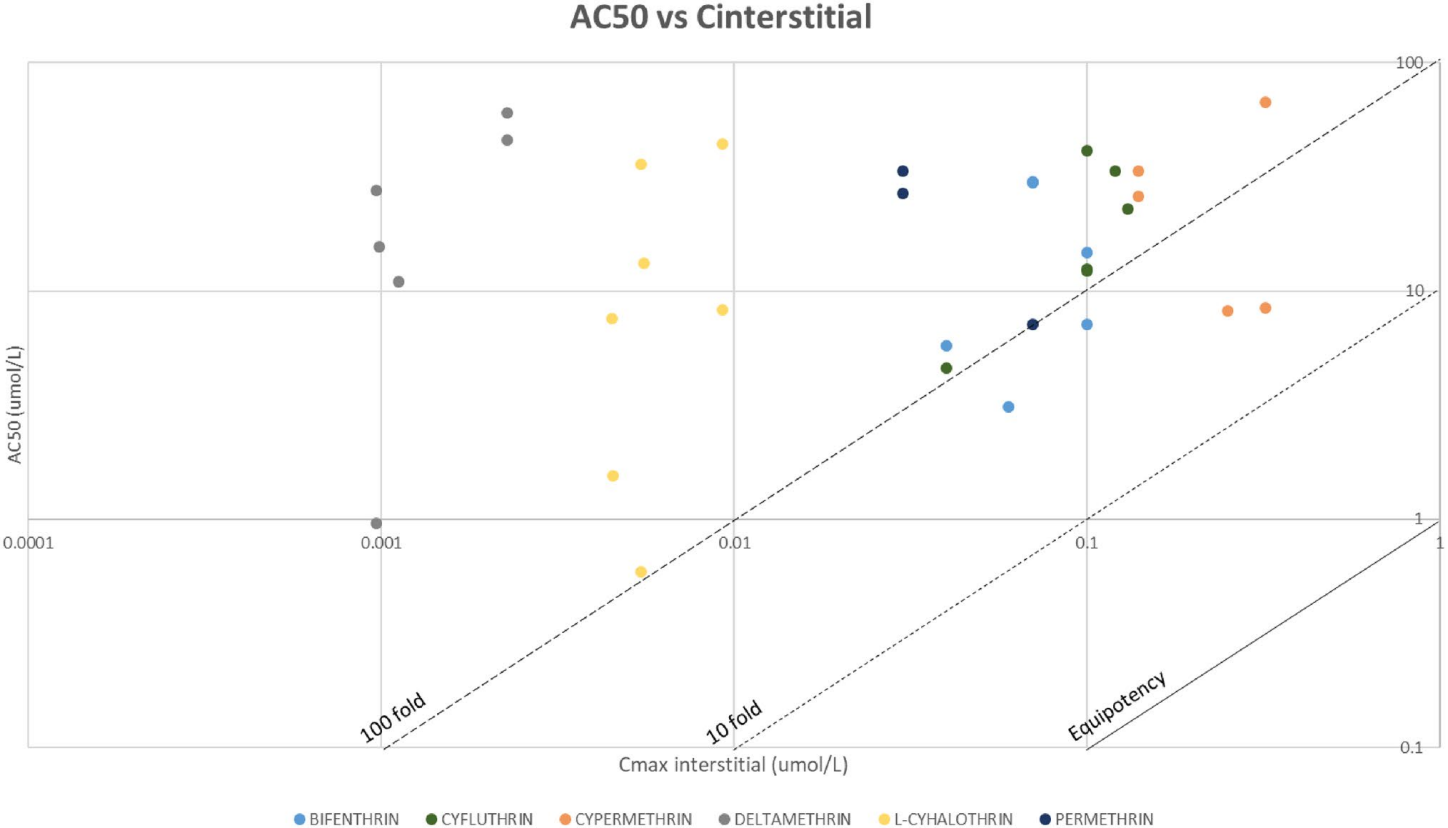
Graph comparing AC_50_ (uM) vs. C_interstitial_ (umol/L). Graph represented in logarithmic scale. The equipotency, the 10-fold and 100-fold lines have been added

**Fig. 7:**
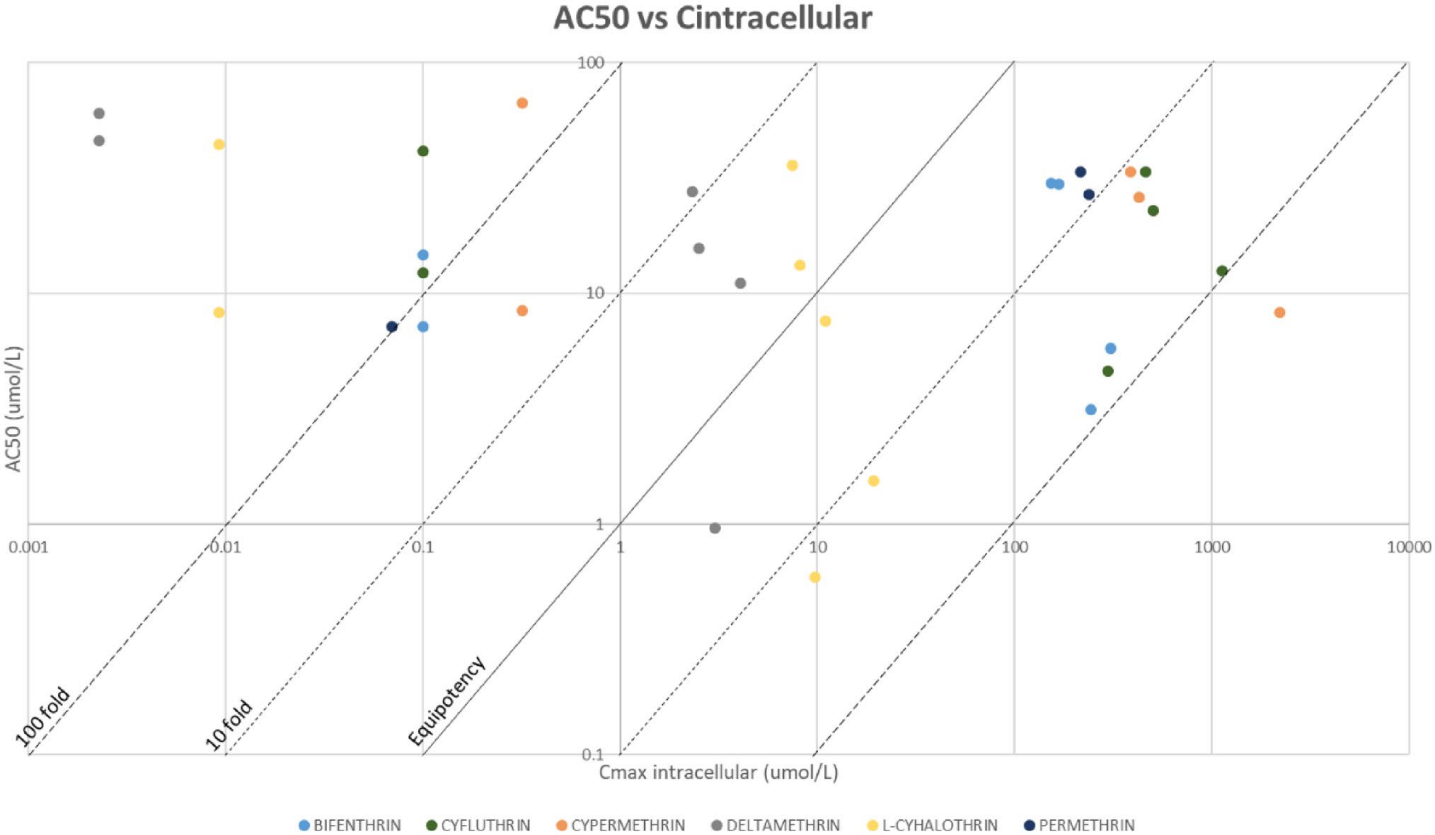
Graph comparing AC_50_ (uM) vs. C_intracellular_ (umol/L). Graph represented in logarithmic scale. The equipotency, the 10-fold and 100-fold lines have been added

Additional simulations were performed using PKSim, to estimate the internal concentrations associated with the tissue/organ NOAEL values for each pyrethroid (Table 9). Comparative graphs were generated, to explore the relationship between in vitro bioactivity and internal dose in vivo PoDs, using the interstitial concentrations (Fig. 6), and the intracellular concentrations (Fig. 7).

**Table 9:** Results from PKSim simulations using NOAEL values: displaying the maximum concentration (C_max_) for each compound in the respective tissues

C_MAX_ (UMOL/L)	Peripheral—general	Brain – neuro repeated c_interst_	Brain – neuro repeated c_intracel_	Kidney – long term c_interst_	Kidney – long term c_intracel_	Liver – long term c_interst_	Liver – long term C_INTRACEL_	Lung – long term C_INTERST_	Lung – long term C_INTRACEL_	Skin – short term C_INTERST_	Skin – short term C_INTRACEL_
Bifenthrin	0.1	0.06	243.15	0.07	152.37	0.07	167.96	0.07	198.59	0.04	306.53
Cyfluthrin	0.1	0.04	296.29	0.12	458.76	0.13	502.24	0.12	598.14	0.1	1129.59
Cypermethrin	0.32	0.25	2209.14	0.14	386.17	0.14	427.29	0.14	503.03	0.22	1914.86
Deltamethrin	2.28E-3	1.12E-3	4.09	9.71E-4	2.32	9.88E-4	2.52	9.71E-4	3.01	–	
L-cyhalothrin	9.28E-3	4.51E-3	10.96	5.45E-3	7.49	5.56E-3	8.17	5.46E-3	9.77	4.52E-3	19.26
Permethrin	0.07	–		0.03	214.29	0.03	236.79	0.03	279.49	–

Based on nominal concentrations, bioactivity is only observed at much higher concentrations than those estimated for interstitial compartments at exposure levels equivalent to the NOAEL. However, when considering intracellular concentrations, the data exhibits a wide distribution. The results triggered the refinement of the bioactivity indicators.

#### Refinement of bioactivity indicators

The refinement modeled freely dissolved (C_free_), cellular (C_cell_), and membrane-bound concentrations (C_mem_) using the Tox21 GeneBLAzer bioassays on a range of neutral and ionogenic organic chemicals with diverse physicochemical characteristics. Since the LSER prediction lacked information for the substances cyfluthrin, deltamethrin and l-cyhalothrin, an extrapolation was carried out from the partition coefficients to obtain the required values (log DBSA/w and log Dlip/w [L/L], pH 7.4, 37 °C). The following results were obtained (Table 10):

**Table 10:** Comparison table from the PKsim vs. the Tox21 GeneBLAzer results. The C_interstitial_ and C_intracellular_ columns are the concentrations obtained from the previous PKsim simulation. The C_norm_ has been stablished with the AC_50_s of each of the tissues of study. The C_free_, C_cell_, C_mem_ are the concentrations obtained from the Tox21 GeneBLAzer estimations

Compound & Target tissue	(E)C_nom_ [umol/L]	C_interst_ (PKsim) [umol/L]	C_intracel_ (Pksim) [umol/L]	(E)C_free_ [umol/L]	(E)C_cell_ [umol/L]	(E)C_mem_ [umol/L]
Bifenthrin						
Vascular system	7.14	0.1	0.1	0.00269	302	11,000
Inmune system	14.7	0.1	0.1	0.00554	621	22,600
Brain	3.12	0.06	243.15	0.00118	132	4800
Kidney	30.09	0.07	152.37	0.0113	1270	46,200
Liver	29.75	0.07	167.96	0.0112	1260	45,700
Skin	5.78	0.04	306.53	0.00218	244	8870
Cyfluthrin						
Vascular system	12.23	0.1	0.1	0.0026*	636*	25,900*
Inmune system	41.3	0.1	0.1	0.0088*	2150*	87,600*
Brain	4.6	0.04	296.29	0.00098*	239*	9750*
Kidney	33.44	0.12	458.76	0.0071*	1740*	70,900*
Liver	22.82	0.13	502.24	0.0048*	1190*	48,400*
Skin	12.42	0.1	1129.59	0.0026*	646*	26,400*
Cypermethrin						
Vascular system	8.43	0.32	0.32	0.00279	379	14,300
Inmune system	66.95	0.32	0.32	0.0222	3010	114,000
Brain	8.21	0.25	2209.14	0.00272	369	13,900
Kidney	33.56	0.14	386.17	0.0111	1510	57,000
Liver	26.03	0.14	427.29	0.00862	1170	44,200
Deltameyhrin						
Vascular system	45.66	0.0023	0.0023	0.016*	1980*	73,100*
Inmune system	60.38	0.0023	0.0023	0.022*	2620*	96,700*
Brain	11.03	0.0011	4.09	0.004*	478*	17,700*
Kidney	27.49	0.00097	2.32	0.0099*	1190*	44,000*
Liver	15.57	0.00099	2.52	0.0056*	675*	24,900*
Lung	0.96	0.00097	3.01	0.00034*	41.6*	1540*
L-Cyhalothrin						
Vascular system	8.25	0.0093	0.0093	0.0048*	250*	6750*
Inmune system	44.09	0.0093	0.0093	0.026*	1330*	36,100*
Brain	7.6	0.0045	10.96	0.0044*	230*	6230*
Kidney	35.9	0.0055	7.49	0.021*	1090*	29,400*
Liver	13.19	0.0056	8.17	0.0077*	399*	10,800*
Lung	0.59	0.0055	9.77	0.00034*	17.9*	483*
Skin	1.55	0.0045	19.26	0.0009*	46.8*	1270*
Permethrin						
Vascular system	7.14	0.07	0.07	0.000372	396	16,600
Kidney	33.615	0.3	214.29	0.00175	1860	78,200
Liver	26.84	0.3	236.79	0.0014	1490	62,500

*LSER prediction lacked information for these compounds. log DBSA/w and log Dlip/w values obtained from extrapolation for Tox21 GeneBLAzer simulation

**Fig. 8:**
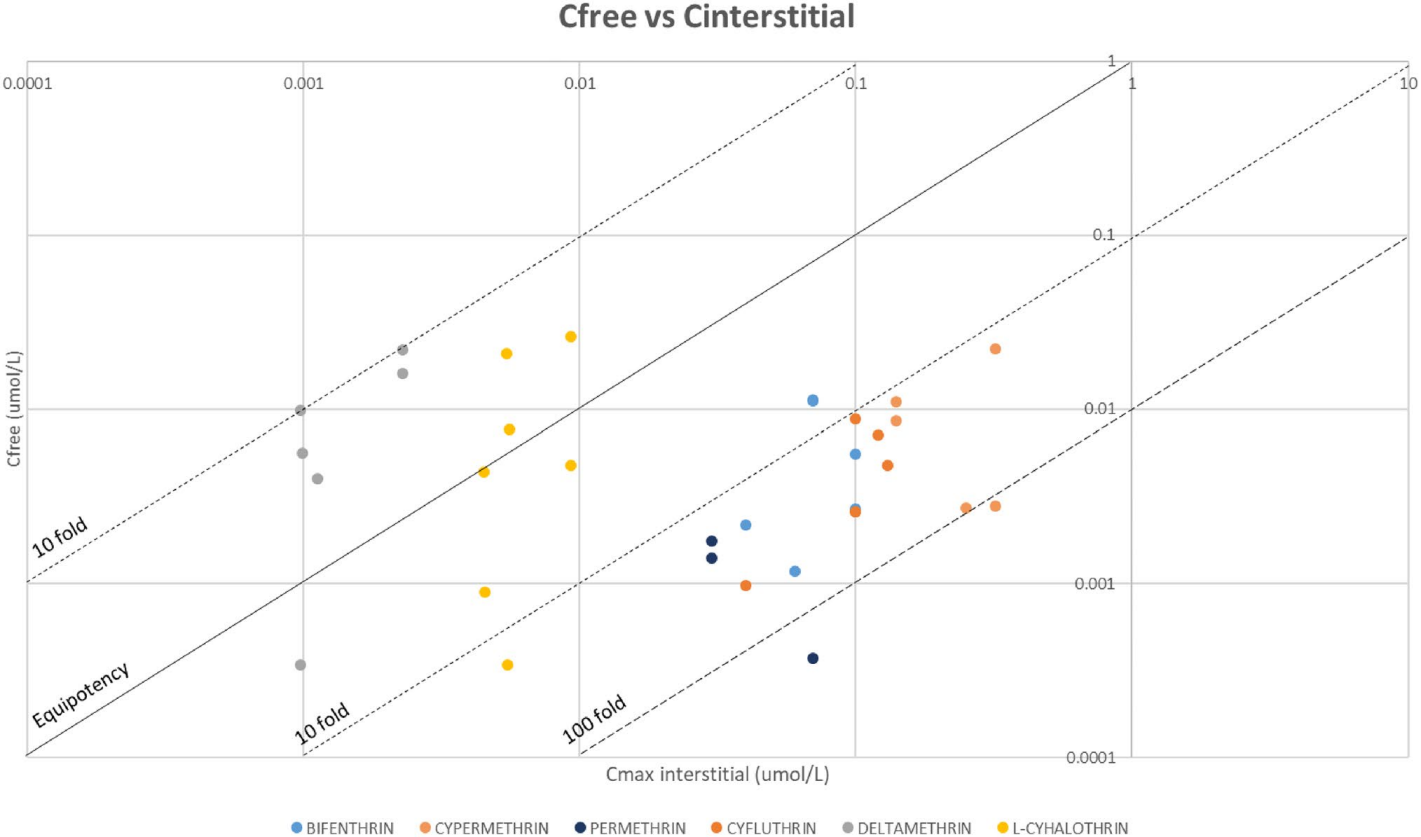
Graph comparing C_free_ (umol/L) vs. C_interstitial_ (umol/L). Graph represented in logarithmic scale. The equipotency, the 10-fold lines have been added. No 100-fold line was required

**Fig. 9:**
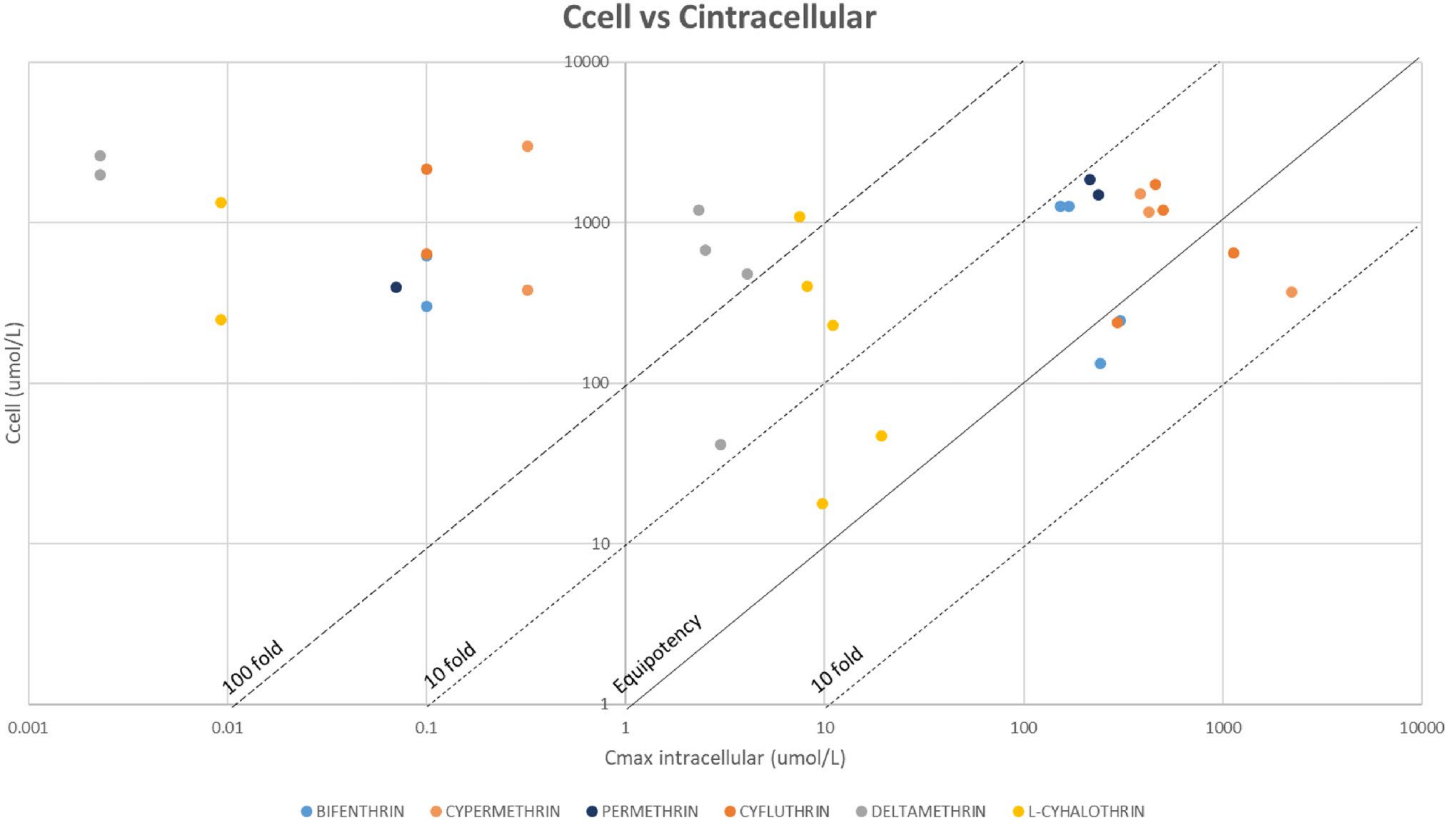
Graph comparing C_cell_ (umol/L) vs. C_intracellular_ (umol/L) - vascular and immune concentrations adapted. Graph represented in logarithmic scale. The equipotency, the 10-fold and 100-fold lines have been added

The relationship between refined in vitro bioactivity and internal dose in vivo PoDs, using the free and interstitial concentrations (Fig. 8), and the intracellular concentrations (Fig. 9) were estimated.

It can be observed that the comparisons for extracellular levels are grouped by pyrethroid, deltamethrin and l-cyhalothrin bioactivity indicators are within a factor of 10 around the equipotency line (Fig. 8) while bifenthrin, cypermethrin, cyfluthrin and permethrin bioactivity indicators are mostly 10 to 100 times lower than the in vivo PoDs. The comparison is less clear for the intracellular concentrations, with most values above the equipotency line.

### Tier 5: Combined RA based on refined bioactivity indicators

Based on the results from the previous tiers, the final risk characterization was based on the refined bioactivity indicators and supported by a comparison with internal dose organ specific in vivo PoDs. Individual and combined MoEs were calculated (Table 11). All MoEs were above 1, but the combined MoE for the vascular system was below 10 and combined MoEs close to 10 were observed for several organs.

**Table 11:**
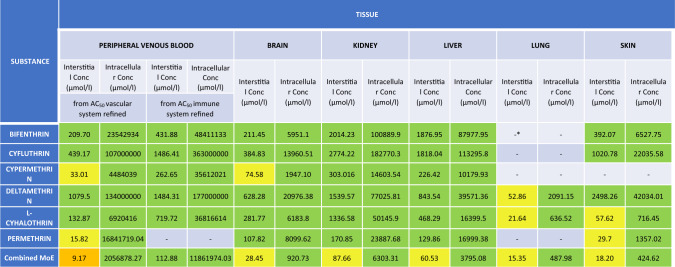
Margin of Exposure (MoE) for tissue-specific bioactivity after refinement. RA characterization for bifenthrin, cyfluthrin, cypermethrin, deltamethrin, l-cyhalothrin, permethrin at realistic exposure levels (de Alba-Gonzalez et al., 2023). Each cell represents the MoE (range between the refined tissue toxicity endpoint by Tox21 and the refined internal exposure predicted by the TK model) of a specific compound in a particular tissue. The color of each cell indicates their probability of producing bioactivation of the risk drivers according to the following code: Red (100). Lastly, a combined MoE was calculated

*Exposure range not calculated due to the lack of AC_50_ bioactivity values.

The parallel assessment based on internal dose in vivo PoDs (Table 12), shows all suggested less concerns, with all individual MoEs higher than 100 and the combined MoEs close to this value.

**Table 12:**
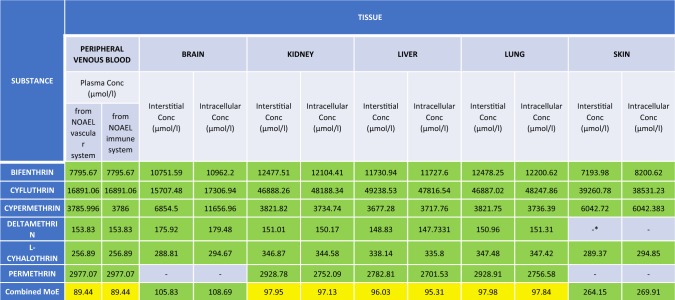
Margin of Exposure (MoE) for tissue-specific bioactivity (in vivo/in vivo). RA characterization for bifenthrin, cyfluthrin, cypermethrin, deltamethrin, l-cyhalothrin, and permethrin at realistic exposure levels (de Alba-Gonzalez et al., 2023). Each cell represents the MoE (range between the tissue toxicity endpoint predicted by the Tk model and the internal exposure also predicted by the TK model) of a specific compound in a particular tissue. The color of each cell indicates their probability of producing bioactivation of the risk drivers according to the following code: Red (100). Lastly, a combined MoE was calculated

*Exposure range not calculated due to the lack of NOAELs in the European Guidelines.

## Discussion

The application of the NGRA framework, particularly the methodology of integrating TKs with NAMs for TDs, offers a transformative strategy for enhancing the regulatory evaluation of chemicals, including pyrethroids. By addressing the limitations of traditional RA practices (Sewell et al. 2024; Lu et al. 2025), NGRA facilitates a more refined evaluation of combined exposures, enabling a nuanced understanding of risks across multiple tissues and exposure scenarios. Pyrethroids provide a compelling case study due to their widespread use, common neurotoxicity concerns with diverse toxicological profiles, and the need for a refined risk assessment of the combined risk of pyrethroid exposure identified in previous assessments (Tarazona et al. 2022).

Human biomonitoring has confirmed pyrethroids among the most relevant pesticides regarding exposure levels in the general population (Andersen et al. 2022a, b) and realistic exposure levels in European adults are available from previous studies under the EU project HBM4EU (VITO HBM 2021) and have been used in previous risk assessments (Tarazona et al. 2022; de Alba et al. 2023).

The proposed framework integrates hypothesis-driven NGRA with a tiered approach, frequently used in (regulatory) risk assessments (Ball et al. 2022). To evaluate the applicability of a NAM-based assessment, a parallel evaluation based on results from in vivo toxicological endpoints extracted from regulatory assessment is included.

In the initial tier, following the bioactivity data extractions leveraging high-throughput data, the critical element is the selection of the bioactivity indicators. As the case study design focused on the use of existing information, the available studies are not the same for all pyrethroids. The proposed way forward is to average the available AC_50_ values grouped by tissue and mechanistic pathways. The integration of results for several related high-throughput studies has been frequently used, e.g. for the screening of endocrine activity by the USEPA (Judson et al. 2015). Several options can be used for the integration of the results from different studies, in our approach we selected the average for each tissue and pathway as a simplistic screening approach.

Considering the similarity in chemical structures and that all pyrethroids are neurotoxicants but with different potencies, Tier 2 explored the possibility for using relative potencies as a standard approach in toxicology (Dinse et al. 2011). The bioactivity results indicated different modes of action with different relative potencies, which was confirmed by the extraction of in vivo organ-specific NOAELs. Consequently, the option for using the toxicity equivalent factor model (Putzrath 1997), was rejected and after assessing options for the problem formulation for combined risk assessments using NAMs (Solomon et al. 2016), the use of MoEs, also proposed by other authors was selected (Nicolas et al. 2022). While interstitial MoE values generally exceeded 100 in the Tier 3 study, intracellular MoE values often fell below this threshold, raising concerns about potential tissue-specific risks (EFSA 2023). An additional analysis was conducted on compounds with MoEs below 100 to investigate the underlying mechanisms affecting tissues and genes showing significant activity. This revealed limitations in the in vitro sensitivity and the necessity of refining bioactivity indicators (Bell et al. 2018), since nominal concentrations did not align with observed bioactivity. Tissue-specific evaluations of compounds with low MoE values identified critical mechanistic pathways driving risk, emphasizing tissue-specific drivers relevant to the combined risk assessments.

The bioactivity indicators based on nominal concentrations identified earlier, were refined to estimate realistic in vitro exposure levels and expected intracellular concentrations and compared with in vivo data in tier 4. The approach considered the importance of incorporating refined toxicokinetics into predictive models to bridge gaps between in vitro and in vivo data (Kenna & Uetrecht 2018). The refinement of in vitro exposure levels has been considered an essential element for improving IVIVE (Henneberger et al. 2021) and NGRA (Nicol et al. 2024), facilitating the assessment of whether variations in toxicity between platforms were attributable to cell line sensitivity, receptor expression, or bioavailability differences (Fischer et al. 2017). The comparison of in vitro bioactivity and the internal dose in vivo PoDs should account interspecies differences, and assessing concordance is a challenge (Lu et al. 2024). Our approach focused on exploratory assessments and the understanding of the observed similarities and differences and their implications in terms of risk assessment. The approach revealed nuanced differences in sensitivity between methods as expected from the different endpoints and biological situations defining the animal and the in vitro models. The aim was to check the capacity of the bioactivity indicators, highlighting areas where in vitro methods offered higher sensitivity and areas where in vivo methods were more reliable. The results underscored the need for continued refinement to improve intracellular concentration estimations and their interpretation.

Finally, two combined MOE calculations were performed as the final and more complex tier of this study: (1) an in vivo*-*in vitro MOE analysis using refined AC_50_ values and (2) an in vivo*-*in vivo MOE analysis using NOAEL values (Djekic et al. 2024; Crivellente et al. 2019). Both approaches indicated safe exposure levels across tissues and compartments, confirming that dietary exposure in healthy male adults does not present significant risks under the studied conditions according to the guideline thresholds levels.

Results indicated that in vivo approaches could be sufficient to confirm a low level of concern for individual exposure levels and a combined risk for dietary exposure below but close to the levels of concern, in line with the comparisons based on internal doses conducted in this study, and previous assessments combining human biomonitoring with traditional risk assessments based on animal models (Tarazona et al. 2022; de Alba et al. 2023). It is important to notice that vascular systems in the in vitro combined MoE analysis demonstrated some potential for exceeding the bioactivity thresholds. Together, these methods offer a comprehensive framework for combined risk assessment of pyrethroids, addressing both systemic and tissue-specific effects. Considering that in the EU there are around 30 different pyrethroids authorized under different regulatory frameworks, the results confirm the need for further assessing the risk associated with the aggregated exposure (US EPA 2001; Price et al. 2020) to pyrethroids from routes other than dietary exposure, with a focus on occupational exposure and groups with expected high exposure due to biocidal and pharmaceutical uses.

This study demonstrates the transformative potential of the use of NAMs for NGRA, particularly those integrating TKs with NAM-based TDs addressing the limitations of the traditional risk assessment (Magurany et al. 2023). By leveraging in silico tools such as PKSim and bioactivity data from high-throughput programs like ToxCast (Wetmore et al. 2015), the approach shifts the focus from traditional reliance on external exposure limits (e.g., ADIs) (Chłodowska et al. 2024) to a more precise evaluation based on internal dose–response relationships.

Unlike conventional risk assessment methodologies, which rely on default extrapolation models and generalized ADI thresholds, the tiered NGRA framework employed in this study facilitates a detailed exploration of cumulative risks associated with combined pyrethroid exposure (Tsai et al. 2023). By incrementally increasing assessment complexity, the approach systematically integrates toxicokinetics via PBPK modeling and bioactivity indicators, enhancing regulatory relevance and practical applicability (Carramusa et al. 2024; Marx-Stoelting et al. 2023).

### Uncertainties and limitations

Any study based on in vitro PoDs has inherent uncertainties and limitations that must be acknowledged (Lu et al. 2024). These arise from both the methodological constraints of in vitro approaches (Motta et al. 2024; Bean et al. 2023) and the specific design of the study. One of the main limitations is the reliance on a specific bioactivity data source, ToxCast, based on a battery of high throughput assays. This limitation was intentional in the design of this proof-of-concept study, as the aim was to focus on publicly available and easily searchable information to check the applicability of the proposed framework in the regulatory context. The results confirm the capacity of the tiered approach to assess the risk of combined exposure. The proposed tiered approach could facilitate further refined when needed, in particular facilitating the design of systematic reviews, or as a first step in an Integrated Approach to Testing and Assessment (IATA) minimizing the need for animal testing (OECD 2017; Casati 2018). Another uncertainty is related to the selection of realistic exposure levels. Dietary exposure depends on the diet and the prevalence and concentrations of each food item, presenting high variability at population level and temporal variability within individuals. The need for consistent criteria for evaluating exposure data sets to represent real-world conditions has been highlighted (Hladik et al. 2024); in our case we used standardized approaches developed by EFSA for prospective estimations, contrasted with HBM4EU harmonized measured human biomonitoring data. Both approaches confirm a significant variability among individuals. It can be argued that the selected value, the middle-bound for PRIMO, does not represent a worst case, and in fact the comparison with human biomonitoring confirms that for the highest exposed population, is between the 75th and 90th percentile; however as human biomonitoring covers all exposure routes, the selected value represents, in our view, a good representation of realistic dietary exposures. This limitation does not affect the comparison of risk assessments based on in vitro bioactivity *vs. *in vivo animal models, as both are based on the same exposure conditions.

For the in vitro component, in addition to focus exclusively on ToxCast data without conducting a systematic review, we selected simple bioindicators, aggregating information from different assays grouped by categories based on information also searchable in ToxCast. The use of average AC_50_ values per category was selected as an easy implementable approach, not requiring expert judgement, to reduce the influence of nonspecific effects, an element that should be considered when using ToxCast data (Escher et al. 2020). There are inherent limitations to the categorization by tissue in ToxCast, as the cell origin does ensure that the functionality of the cell type under in vivo conditions is maintained in vitro. The use of aggregated data, the specific assessment under Tier 3, and the approach for presenting MoEs for all categories through the full process were introduced in the framework for minimizing the consequences of this limitation. Future research should explore the most appropriate bioindicators for comparing in vivo and in vitro data to enhance accuracy and regulatory acceptance.

For the in vivo analysis, we only used summary evaluations from EFSA and ECHA. This restricts the depth of the analysis but, on the other hand, it allows the study to align with regulatory assessment frameworks, where risk assessments often rely on existing evaluations (Reffstrup et al. 2010). Similarly, in our TK assessments, we used generic, publicly available models and existing data, maximizing the use of default parameters and exposure scenarios. This facilitates regulatory applicability but introduces uncertainties since compound-specific refinements were not applied (Thépaut et al. 2024). The MoEs based on in vivo animal models are based on TK simulations conducted with the same model and parameters, this is expected to reduce the uncertainty as in the extrapolation from external to internal doses in the exposure and effect estimations used the same approach, consequently, although the PoDs were selected from animal models, the estimation of the corresponding internal doses was for humans exposed at the selected PoD level.

The situation is more complex for the in vitro data, as the model used for the refinement has different approaches, e.g., accounting for protein binding, than the TK model used for the internal exposure estimations. Considering these limitations and the inherent differences between the in vitro models and the in vivo situation, the selected risk characterization approach is through the estimation of independent MoEs for complementary PoDs. This allows specific considerations for addressing the uncertainties in the categorization of the MoE’s ranges and facilitates prioritization.

Considering the use of different tools and the limitations, the approach for the in vitro-in vivo comparisons has been to explore different options, assessing both similarities and differences as global trends instead of specific cases. The assessments are based on different models, species, endpoints, levels of biological organization, etc. In addition, animal studies are just “a model” for estimating risk for humans. Consequently, the proposed approach for the comparison is based on the “coherence” in the interpretation of the results, considering the context of each set of data. Again, the uncertainty is considered in the categorization of the MoEs, with risk categories adapted to the specific data context.

## Conclusions

A key advancement of this study is the application of a tiered NGRA framework that refines the evaluation of combined exposures. The proposed framework assesses complementary estimations for different bioactivity indicators, grouped by pathway and tissue, through a tiered process for addressing the risk associated with combined exposures. The capability of the bioactivity-based approach for assessing the combined risk of pyrethroids has been confirmed through a parallel assessment using traditional animal studies.

The bioactivity assessment was sufficient to conclude that the approach of relative potency for addressing combined risk is not applicable in the case of pyrethroids. As expected, this conclusion was confirmed by the parallel in vivo assessment.

Regarding the risk characterization approach, the tiered evaluations within NGRA further validate the utility of MoE calculations in risk characterization (Doménech & Martorell 2024), including combined exposure scenarios. The MoE approach facilitates the comparison of risk characterization outcomes based on different models and approaches, as well as the incorporation of context-related uncertainties through the categorization of MoE ranges adapted to the specific assessment conditions. In this proof-of-concept study, the approach of parallel assessment lines based on complementary PoDs and a combined MoE for each assessment line has been also applied to the animal models, addressing the different relative potencies also observed in the in vivo studies. In this context, the MoE approach allows for risk characterization without relying on strict thresholds, and the combination of MoEs enables the estimation of cumulative exposure, which is crucial in regulatory risk assessment.

For pyrethroids, while dietary exposure seems to be close to but below concerning levels, additional exposure sources could bring risk estimates of potential concern for certain population groups, requiring further assessments of aggregate exposure.

In conclusion, this study confirms the value of NGRA as a scalable, robust, and regulatory-aligned framework for chemical risk assessment. The integration of TK data into a multi-tiered approach facilitates a dynamic understanding of metabolic and toxicity processes over time. This methodological innovation not only confirms the validity of this risk assessment approach for pyrethroids but also establishes a transferable model applicable to other chemical classes with complex exposure and toxicity profiles. Finally, the methodology’s scalability and accessibility, being cost-effective and aligned with current evaluation processes, make it a viable candidate for inclusion in regulatory guidelines and models representing a great methodology for safety evaluation and risk assessment, paving the way for improved regulatory standards and public health protections.

## Supplementary Information

Below is the link to the electronic supplementary material.

Supplementary file1 (DOCX 185 KB)

## Data Availability

This study utilized publicly available datasets from the ToxCast database, accessible at https://comptox.epa.gov/dashboard/. Please cite the original dataset as: CompTox Chemicals Dashboard (2024). Epa.gov. The other source of data was the ECHA and EFSA conclusions from each of the studied Pyrethroids (sources referenced in this manuscript).

## Declarations

### Human and animal studies

The research utilized publicly available data only, and no new human or animal studies were conducted.
