# Supplementary material for: A tiered next-generation risk assessment framework integrating toxicokinetics and NAM-based toxicodynamics: “proof of concept” case study using pyrethroids
Source: Arch Toxicol. 2025 May 7;99(7):2759–81. doi: 10.1007/s00204-025-04045-9 (PMC12198285; doi:10.1007/s00204-025-04045-9)
Supplement: Supplementary file 1 — Supplementary file1 (DOCX 185 KB) [file 204_2025_4045_MOESM1_ESM.docx]

# Appendix 1:

**Table S1:** **Bifenthrin´s ToxCast information on the tissue categories**. Extracted information of Bifenthrin on its tissue and the corresponding AC50s extracted from the ToxCast database. The simplified tissue categories correspond to the categories given by the author for the purposes of this study.

| *TOXCAST BIOLOGICAL PROCESS TARGET* | *TOXCAST TISSUE* | *TOXCAST AC50 VALUE (uM)* | *Simplified TISSUE CATEGORY* |
| --- | --- | --- | --- |
| *Transcription Factor Activity* | liver | 19.95 | Liver |
| *Functional Neural Network Activity* | cortical | 2.33 | Brain |
| *Cell Proliferation* | prostate | 66.22 | Prostate function |
| *Functional Neural Network Activity* | cortical | 0.79 | Brain |
| *Mrna - Cyp* | liver | 4.01 | Liver |
| *Spontaneous Neural Activity* | cortical | 1.65 | Brain |
| *Transcription Factor Activity* | kidney | 23.04 | Kidney |
| *Mrna - Cyp* | liver | 3.86 | Liver |
| *Gene Expression* | skin | 2.48 | Skin |
| *Dna Repair* | lymphoblast | 11.32 | Immune system |
| *Functional Neural Network Activity* | cortical | 0.23 | Brain |
| *Transcription Factor Activity* | liver | 47.3 | Liver |
| *Transcription Factor Activity* | liver | 29.35 | Liver |
| *Cell Proliferation* | lymphoblast | 8.93 | Immune system |
| *Gene Expression* | vascular | 4.12 | Vascular system |
| *Spontaneous Neural Activity* | cortical | 1.47 | Brain |
| *Cell Proliferation* | kidney | 74.95 | Kidney |
| *Spontaneous Neural Activity* | cortical | 2.29 | Brain |
| *Transcription Factor Activity* | liver | 100 | Liver |
| *Transcription Factor Activity* | liver | 100 | Liver |
| *Transcription Factor Activity* | liver | 34.1 | Liver |
| *Cell Proliferation* | kidney | 21.79 | Kidney |
| *Transcription Factor Activity* | liver | 34.6 | Liver |
| *Gene Expression* | vascular | 4.49 | Vascular system |
| *Functional Neural Network Activity* | cortical | 0.09 | Brain |
| *Protein Stabilization* | kidney | 73.11 | Kidney |
| *Transcription Factor Activity* | liver | 76.06 | Liver |
| *Transcription Factor Activity* | liver | 12.51 | Liver |
| *Mrna - Cyp* | liver | 33.82 | Liver |
| *Transcription Factor Activity* | liver | 12.22 | Liver |
| *Transcription Factor Activity* | kidney | 7.94 | Kidney |
| *Functional Neural Network Activity* | cortical | 1.51 | Brain |
| *Gene Expression* | vascular | 2.87 | Vascular system |
| *Transcription Factor Activity* | liver | 50 | Liver |
| *Cell Proliferation* | intestinal | 26.84 | Intestine |
| *Cell Proliferation* | vascular | 9.6 | Vascular system |
| *Gene Expression* | vascular | 6.88 | Vascular system |
| *Cell Proliferation* | kidney | 23.66 | Kidney |
| *Spontaneous Neural Activity* | cortical | 0.76 | Brain |
| *Cell Death* | vascular | 8.66 | Vascular system |
| *Transcription Factor Activity* | liver | 26.66 | Liver |
| *Functional Neural Network Activity* | cortical | 0.14 | Brain |
| *Transcription Factor Activity* | liver | 24.66 | Liver |
| *Cell Death* | cortical | 14.21 | Brain |
| *Spontaneous Neural Activity* | cortical | 2.8 | Brain |
| *Gene Expression* | vascular | 3.95 | Vascular system |
| *Transcription Factor Activity* | liver | 2.44 | Liver |
| *Transcription Factor Activity* | liver | 100 | Liver |
| *Transcription Factor Activity* | liver | 49.23 | Liver |
| *Mrna - Cyp* | liver | 3.25 | Liver |
| *Transcription Factor Activity* | liver | 1.64 | Liver |
| *Gene Expression* | skin | 3.97 | Skin |
| *Spontaneous Neural Activity* | cortical | 3.23 | Brain |
| *Spontaneous Neural Activity* | cortical | 2.84 | Brain |
| *Transcription Factor Activity* | liver | 37.1 | Liver |
| *Transcription Factor Activity* | liver | 0.7 | Liver |
| *Transcription Factor Activity* | liver | 20.81 | Liver |
| *Cell Proliferation* | vascular | 10.35 | Vascular system |
| *Catalytic Activity* | NA | 13.02 | - |
| *Cell Proliferation* | prostate | 22.02 | Prostate function |
| *Transcription Factor Activity* | liver | 10.47 | Liver |
| *Receptor Binding* | NA | 0.33 | - |
| *Cell Proliferation* | Ovary | 70.57 | Ovary |
| *Cell Proliferation* | prostate | 29.95 | Prostate function |
| *Transcription Factor Activity* | pituitary gland | 17.73 | Brain |
| *Cell Morphology* | vascular | 0.1 | Vascular system |
| *Functional Neural Network Activity* | cortical | 0.44 | Brain |
| *Mrna - Cyp* | liver | 32.8 | Liver |
| *Transcription Factor Activity* | liver | 3.66 | Liver |
| *Transcription Factor Activity* | liver | 100 | Liver |
| *Gene Expression* | vascular | 9.23 | Vascular system |
| *Cytotoxicity* | Ovary | 39.32 | Ovary |
| *Transcription Factor Activity* | liver | 75.11 | Liver |
| *Transcription Factor Activity* | breast | 45 | Breast |
| *Cell Death* | cortical | 15.8 | Brain |
| *Cell Death* | vascular | 8.88 | Vascular system |
| *Transcription Factor Activity* | liver | 5.56 | Liver |
| *Transcription Factor Activity* | liver | 31.74 | Liver |
| *Transcription Factor Activity* | kidney | 20.35 | Kidney |
| *Gene Expression* | vascular | 3.85 | Vascular system |
| *Functional Neural Network Activity* | cortical | 0.93 | Brain |
| *Transcription Factor Activity* | liver | 88.29 | Liver |
| *Spontaneous Neural Activity* | cortical | 1.56 | Brain |
| *Transcription Factor Activity* | liver | 12.19 | Liver |
| *Mrna - Cyp* | liver | 20 | Liver |
| *Spontaneous Neural Activity* | cortical | 0.85 | Brain |
| *Mrna - Cyp* | liver | 7.83 | Liver |
| *Gene Expression* | vascular | 5.12 | Vascular system |
| *Transcription Factor Activity* | liver | 12.62 | Liver |
| *Transcription Factor Activity* | liver | 19.84 | Liver |
| *Mrna - Cyp* | liver | 5.45 | Liver |
| *Dna Repair* | lymphoblast | 23.85 | Immune system |
| *Transcription Factor Activity* | kidney | 14.24 | Kidney |
| *Gene Expression* | skin | 3.97 | Skin |
| *Cell Proliferation* | kidney | 40 | Kidney |
| *Mrna - Cyp* | liver | 20 | Liver |
| *Transcription Factor Activity* | liver | 14.16 | Liver |
| *Cell Proliferation* | Ovary | 100 | Ovary |
| *Transcription Factor Activity* | liver | 34.39 | Liver |
| *Transcription Factor Activity* | kidney | 13.31 | Kidney |
| *Transcription Factor Activity* | liver | 14.36 | Liver |
| *Cell Proliferation* | kidney | 23.9 | Kidney |
| *Cell Proliferation* | liver | 45 | Liver |
| *Transcription Factor Activity* | breast | 59.48 | Breast |
| *Cell Proliferation* | skin | 12.68 | Skin |
| *Mrna - Cyp* | liver | 33.89 | Liver |
| *Transcription Factor Activity* | liver | 42.26 | Liver |
| *Gene Expression* | vascular | 13.21 | Vascular system |
| *Gene Expression* | vascular | 7.34 | Vascular system |
| *Cell Proliferation* | kidney | 24.8 | Kidney |
| *Transcription Factor Activity* | liver | 1.81 | Liver |
| *Gene Expression* | vascular | 15.54 | Vascular system |
| *Transcription Factor Activity* | liver | 4.74 | Liver |
| *Functional Neural Network Activity* | cortical | 2.29 | Brain |
| *Mrna - Cyp* | liver | 4.42 | Liver |
| *Functional Neural Network Activity* | cortical | 0.39 | Brain |
| *Mitochondrial Depolarization* | liver | 36.64 | Liver |
| *Transcription Factor Activity* | liver | 11.32 | Liver |
| *Functional Neural Network Activity* | cortical | 0.66 | Brain |
| *Spontaneous Neural Activity* | cortical | 0.77 | Brain |
| *Functional Neural Network Activity* | cortical | 2.29 | Brain |
| *Development* | Ovary | 0.53 | Ovary |
| *Mrna - Cyp* | liver | 4.58 | Liver |

**Table S2:** **Bifenthrin´s ToxCast information on the gene categories**. Extracted information of Bifenthrin on its gene activation and the corresponding AC50s extracted from the ToxCast database. The simplified gene category corresponds to the gene categories given by the author for the purposes of this study.

| *TOXCAST GENE SYMBOL* | *TOXCAST AC50 VALUE (uM)* | *SIMPLIFIED GENE CATEGORY* |
| --- | --- | --- |
| *ESR1* | 19.95 | Estrogen |
| *-* | 2.33 | - |
| *-* | 66.22 | - |
| *-* | 0.79 | - |
| *CYP3A4* | 4.01 | Cytochrome |
| *-* | 1.65 | - |
| *ESRRA* | 23.04 | Estrogen |
| *CYP3A4* | 3.86 | Cytochrome |
| *MMP1* | 2.48 | Proteolysis |
| *RAD54L \| XRCC6P1* | 11.32 | DNA |
| *-* | 0.23 | - |
| *CYP2E1* | 47.3 | Cytochrome |
| *ESR1* | 29.35 | Estrogen |
| *-* | 8.93 | - |
| *PTGER2* | 4.12 | Prostaglandin |
| *-* | 1.47 | - |
| *-* | 74.95 | - |
| *-* | 2.29 | - |
| *esr2b* | 100 | Estrogen |
| *ESR2* | 100 | Estrogen |
| *FABP1* | 34.1 | Fatty acid |
| *-* | 21.79 | - |
| *esr1.L* | 34.6 | Estrogen |
| *THBD* | 4.49 | Membrane receptor - Thrombosis |
| *-* | 0.09 | - |
| *ESR1 \| ESR2* | 73.11 | Estrogen |
| *CYP1A2* | 76.06 | Cytochrome |
| *NR1I2* | 12.51 | Nuclear receptor |
| *CYP1A2* | 33.82 | Cytochrome |
| *VDR* | 12.22 | Nuclear receptor |
| *AR* | 7.94 | Androgen receptor |
| *-* | 1.51 | - |
| *CSF1* | 2.87 | Proteolysis |
| *ABCG2* | 50 | ATP binding |
| *-* | 26.84 | - |
| *-* | 9.6 | - |
| *CXCL8* | 6.88 | Inflammation |
| *-* | 23.66 | - |
| *-* | 0.76 | - |
| *-* | 8.66 | - |
| *ABCB1* | 26.66 | ATP binding |
| *-* | 0.14 | - |
| *ESR1* | 24.66 | Estrogen |
| *-* | 14.21 | - |
| *-* | 2.8 | - |
| *HLA-DRA* | 3.95 | Immune system receptor |
| *NR1I2* | 2.44 | Nuclear receptor |
| *esr2.L* | 100 | Estrogen |
| *esr1.L* | 49.23 | Estrogen |
| *CYP2B6* | 3.25 | Cytochrome |
| *NR1I2* | 1.64 | Nuclear receptor |
| *CSF1* | 3.97 | Proteolysis |
| *-* | 3.23 | - |
| *-* | 2.84 | - |
| *ESR1* | 37.1 | Estrogen |
| *NFKB1* | 0.7 | Androgen receptor |
| *ESR1* | 20.81 | Estrogen |
| *-* | 10.35 | - |
| *CYP2C19* | 13.02 | Cytochrome |
| *AR* | 22.02 | Androgen receptor |
| *NR1I3* | 10.47 | Nuclear receptor |
| *SLC6A2* | 0.33 | Neuroreceptor |
| *-* | 70.57 | - |
| *-* | 29.95 | - |
| *THRA \| THRB* | 17.73 | thyroid hormone receptor |
| *-* | 0.1 | - |
| *-* | 0.44 | - |
| *CYP1A1* | 32.8 | Cytochrome |
| *CYP2B6* | 3.66 | Cytochrome |
| *esr2.L* | 100 | Estrogen |
| *SELP* | 9.23 | Protein coding |
| *-* | 39.32 | - |
| *esr2b* | 75.11 | Estrogen |
| *ESR1* | 45 | Estrogen |
| *-* | 15.8 | - |
| *-* | 8.88 | - |
| *ESR1* | 5.56 | Estrogen |
| *CYP1A1* | 31.74 | Cytochrome |
| *PGR* | 20.35 | progesterone receptor |
| *VCAM1* | 3.85 | Membrane receptor - artherosclerosis |
| *-* | 0.93 | - |
| *DDIT3* | 88.29 | DNA |
| *-* | 1.56 | - |
| *UGT1A1* | 12.19 | glucuronidation |
| *UGT1A1* | 20 | glucuronidation |
| *-* | 0.85 | - |
| *CYP3A4* | 7.83 | Cytochrome |
| *CD69* | 5.12 | Proliferation |
| *ESR1* | 12.62 | Estrogen |
| *ESR1* | 19.84 | Estrogen |
| *SULT2A1* | 5.45 | sulfotransferase |
| *REV3L* | 23.85 | DNA |
| *-* | 14.24 | - |
| *MMP9* | 3.97 | Proteolysis |
| *-* | 40 | - |
| *CYP1A1* | 20 | Cytochrome |
| *CYP2C19* | 14.16 | Cytochrome |
| *-* | 100 | - |
| *IGF1* | 34.39 | Insulin |
| *NR1H4* | 13.31 | Nuclear receptor |
| *CYP7A1* | 14.36 | Cytochrome |
| *-* | 23.9 | - |
| *-* | 45 | - |
| *AR* | 59.48 | Androgen receptor |
| *-* | 12.68 | - |
| *ABCB1* | 33.89 | ATP binding |
| *Pparg* | 42.26 | Nuclear receptor |
| *CD40* | 13.21 | Immune system receptor |
| *SELE* | 7.34 | Inflammation |
| *-* | 24.8 | - |
| *NR1I2* | 1.81 | Nuclear receptor |
| *THBD* | 15.54 | Membrane receptor - Thrombosis |
| *CYP3A7* | 4.74 | Cytochrome |
| *-* | 2.29 | - |
| *CYP2B6* | 4.42 | Cytochrome |
| *-* | 0.39 | - |
| *-* | 36.64 | - |
| *CYP3A4* | 11.32 | Cytochrome |
| *-* | 0.66 | - |
| *-* | 0.77 | - |
| *-* | 2.29 | - |
| *-* | 0.53 | - |
| *CYP2B6* | 4.58 | Cytochrome |

**Table S3:** **Cyfluthrin´s ToxCast information on the tissue categories**. Extracted information of Cyfluthrin on its tissue and the corresponding AC50s extracted from the ToxCast database. The simplified tissue categories correspond to the categories given by the author for the purposes of this study.

| *TOXCAST BIOLOGICAL PROCESS TARGET* | | *TOXCAST TISSUE* | *TOXCAST AC50 VALUE (uM)* | | | *Simplified TISSUE CATEGORY* |
| --- | --- | --- | --- | --- | --- | --- |
| *Transcription Factor Activity* | liver | | | 50 | Liver | |
| *Functional Neural Network Activity* | cortical | | | 5.46 | Brain | |
| *Transcription Factor Activity* | liver | | | 36.16 | Liver | |
| *Cell Proliferation* | breast | | | 75.14 | Breast | |
| *Functional Neural Network Activity* | cortical | | | 0.12 | Brain | |
| *Cell Proliferation* | prostate | | | 65.15 | Prostate function | |
| *Cell Proliferation* | skin | | | 10.37 | Skin | |
| *Spontaneous Neural Activity* | cortical | | | 0.41 | Brain | |
| *Spontaneous Neural Activity* | cortical | | | 2.63 | Brain | |
| *Transcription Factor Activity* | kidney | | | 65.12 | Kidney | |
| *Transcription Factor Activity* | cervix | | | 55.46 | Ovary | |
| *Catalytic Activity* | NA | | | 10 | - | |
| *Transcription Factor Activity* | liver | | | 50.46 | Liver | |
| *Functional Neural Network Activity* | cortical | | | 0.17 | Brain | |
| *Gene Expression* | vascular | | | 4.55 | Vascular system | |
| *Dna Repair* | lymphoblast | | | 41.3 | Immune system | |
| *Functional Neural Network Activity* | cortical | | | 0.07 | Brain | |
| *Gene Expression* | vascular | | | 20 | Vascular system | |
| *Gene Expression* | skin | | | 3.09 | Skin | |
| *Transcription Factor Activity* | liver | | | 50 | Liver | |
| *Spontaneous Neural Activity* | cortical | | | 2.04 | Brain | |
| *Transcription Factor Activity* | liver | | | 12.13 | Liver | |
| *Transcription Factor Activity* | liver | | | 50 | Liver | |
| *Spontaneous Neural Activity* | cortical | | | 0.57 | Brain | |
| *Neurodevelopment* | cortical | | | 2.98 | Brain | |
| *Functional Neural Network Activity* | cortical | | | 10 | Brain | |
| *Transcription Factor Activity* | cervix | | | 25.57 | Ovary | |
| *Transcription Factor Activity* | liver | | | 19.03 | Liver | |
| *Gene Expression* | skin | | | 0.1 | Skin | |
| *Spontaneous Neural Activity* | cortical | | | 0.21 | Brain | |
| *Transcription Factor Activity* | liver | | | 50 | Liver | |
| *Cell Death* | vascular | | | 9.14 | Vascular system | |
| *Transcription Factor Activity* | liver | | | 20.59 | Liver | |
| *Cell Proliferation* | vascular | | | 7.79 | Vascular system | |
| *Spontaneous Neural Activity* | cortical | | | 10 | Brain | |
| *Functional Neural Network Activity* | cortical | | | 0.27 | Brain | |
| *Transcription Factor Activity* | liver | | | 25.47 | Liver | |
| *Transcription Factor Activity* | kidney | | | 14.71 | Kidney | |
| *Transcription Factor Activity* | liver | | | 2.76 | Liver | |
| *Steroid Hormone Biosynthetic Process* | adrenal gland | | | 66.46 | Kidney | |
| *Cell Proliferation* | liver | | | 40 | Liver | |
| *Mrna - Cyp* | liver | | | 7.55 | Liver | |
| *Spontaneous Neural Activity* | cortical | | | 0.45 | Brain | |
| *Transcription Factor Activity* | liver | | | 19.84 | Liver | |
| *Transcription Factor Activity* | liver | | | 1.73 | Liver | |
| *Transcription Factor Activity* | liver | | | 8.81 | Liver | |
| *Spontaneous Neural Activity* | cortical | | | 10 | Brain | |
| *Functional Neural Network Activity* | cortical | | | 0.05 | Brain | |
| *Transcription Factor Activity* | liver | | | 50 | Liver | |
| *Spontaneous Neural Activity* | cortical | | | 10 | Brain | |
| *Cell Proliferation* | vascular | | | 29.49 | Vascular system | |
| *Transcription Factor Activity* | liver | | | 0.42 | Liver | |
| *Cell Proliferation* | prostate | | | 23.65 | Prostate function | |
| *Transcription Factor Activity* | liver | | | 9.81 | Liver | |
| *Transcription Factor Activity* | liver | | | 50 | Liver | |
| *Cell Proliferation* | prostate | | | 27.93 | Prostate function | |
| *Cell Morphology* | vascular | | | 7.54 | Vascular system | |
| *Receptor Binding* | NA | | | 2.15 | - | |
| *Catalytic Activity* | NA | | | 13.59 | - | |
| *Transcription Factor Activity* | liver | | | 16.75 | Liver | |
| *Transcription Factor Activity* | liver | | | 16.08 | Liver | |
| *Spontaneous Neural Activity* | cortical | | | 7.7 | Brain | |
| *Transcription Factor Activity* | pituitary gland | | | 39.9 | Brain | |
| *Cell Proliferation* | liver | | | 27.94 | Liver | |
| *Gene Expression* | vascular | | | 32.19 | Vascular system | |
| *Functional Neural Network Activity* | cortical | | | 3.51 | Brain | |
| *Transcription Factor Activity* | liver | | | 19.84 | Liver | |
| *Cell Death* | vascular | | | 4.54 | Vascular system | |
| *Transcription Factor Activity* | liver | | | 10 | Liver | |
| *Functional Neural Network Activity* | cortical | | | 1.72 | Brain | |
| *Transcription Factor Activity* | liver | | | 15.16 | Liver | |
| *Transcription Factor Activity* | cervix | | | 46.17 | Ovary | |
| *Receptor Binding* | kidney | | | 11.83 | Kidney | |
| *Functional Neural Network Activity* | cortical | | | 7.23 | Brain | |
| *Transcription Factor Activity* | liver | | | 32.56 | Liver | |
| *Transcription Factor Activity* | liver | | | 20.7 | Liver | |
| *Mrna - Cyp* | liver | | | 20 | Liver | |
| *Gene Expression* | skin | | | 12.65 | Skin | |
| *Cell Death* | vascular | | | 5.88 | Vascular system | |
| *Transcription Factor Activity* | liver | | | 12.91 | Liver | |
| *Functional Neural Network Activity* | cortical | | | 15 | Brain | |
| *Transcription Factor Activity* | liver | | | 9.94 | Liver | |
| *Catalytic Activity* | NA | | | 3.66 | - | |
| *Spontaneous Neural Activity* | cortical | | | 1.07 | Brain | |
| *Transcription Factor Activity* | kidney | | | 12.01 | Kidney | |
| *Transcription Factor Activity* | liver | | | 19.6 | Liver | |
| *Spontaneous Neural Activity* | cortical | | | 1.15 | Brain | |
| *Functional Neural Network Activity* | cortical | | | 3.15 | Brain | |
| *Transcription Factor Activity* | kidney | | | 23.96 | Kidney | |
| *Receptor Binding* | NA | | | 14.31 | - | |
| *Mitochondrial Depolarization* | liver | | | 44.2 | Liver | |
| *Cell Proliferation* | liver | | | 70.98 | Liver | |
| *Transcription Factor Activity* | liver | | | 2.12 | Liver | |
| *Transcription Factor Activity* | liver | | | 17.23 | Liver | |
| *Spontaneous Neural Activity* | cortical | | | 0.17 | Brain | |
| *Gene Expression* | vascular | | | 6.61 | Vascular system | |
| *Spontaneous Neural Activity* | cortical | | | 2.28 | Brain | |
| *Development* | ovary | | | 0.32 | Ovary | |
| *Gene Expression* | vascular | | | 6.77 | Vascular system | |
| *Cell Death* | skin | | | 35.91 | Skin | |
| *Mrna - Cyp* | liver | | | 7.96 | Liver | |
| *Transcription Factor Activity* | cervix | | | 32.78 | Ovary | |
| *Spontaneous Neural Activity* | cortical | | | 1.66 | Brain | |
| *Neurodevelopment* | cortical | | | 15 | Brain | |
| *Transcription Factor Activity* | liver | | | 7.39 | Liver | |
| *Transcription Factor Activity* | liver | | | 14.27 | Liver | |
| *Functional Neural Network Activity* | cortical | | | 0.23 | Brain | |
| *Transcription Factor Activity* | liver | | | 21.99 | Liver | |
| *Cell Proliferation* | kidney | | | 40 | Kidney | |
| *Transcription Factor Activity* | liver | | | 7.71 | Liver | |
| *Transcription Factor Activity* | liver | | | 8.82 | Liver | |
| *Mrna - Cyp* | liver | | | 20 | Liver | |
| *Transcription Factor Activity* | liver | | | 15.5 | Liver | |
| *Spontaneous Neural Activity* | cortical | | | 0.39 | Brain | |
| *Transcription Factor Activity* | liver | | | 30.07 | Liver | |
| *Functional Neural Network Activity* | cortical | | | 0.1 | Brain | |
| *Receptor Binding* | NA | | | 6.93 | - | |
| *Transcription Factor Activity* | liver | | | 2.41 | Liver | |
| *Transcription Factor Activity* | liver | | | 45.28 | Liver | |
| *Functional Neural Network Activity* | cortical | | | 0.62 | Brain | |
| *Transcription Factor Activity* | liver | | | 3.23 | Liver | |

**Table S4:** **Cyfluthrin´s ToxCast information on the gene categories**. Extracted information of Cyfluthrin on its gene activation and the corresponding AC50s extracted from the ToxCast database. The simplified gene category corresponds to the gene categories given by the author for the purposes of this study.

| TOXCAST GENE SYMBOL | *TOXCAST AC50 VALUE (uM)* | *SIMPLIFIED GENE CATEGORY* |
| --- | --- | --- |
| SLC10A1 | 50 | Membrane receptor - liver |
| - | 5.46 | - |
| thra.L | 36.16 | thyroid hormone receptor |
| - | 75.14 | - |
| - | 0.12 | - |
| - | 65.15 | - |
| - | 10.37 | - |
| - | 0.41 | - |
| - | 2.63 | - |
| ESRRA | 65.12 | Estrogen |
| - | 55.46 | - |
| CYP2C9 | 10 | Cytochrome |
| CYP2E1 | 50.46 | Cytochrome |
| - | 0.17 | - |
| PTGER2 | 4.55 | Prostaglandin |
| RAD54L \| XRCC6P1 | 41.3 | DNA |
| - | 0.07 | - |
| CXCL8 | 20 | progesterone receptor |
| EGFR | 3.09 | Protein kinase |
| PDK4 | 50 | Protein kinase |
| - | 2.04 | - |
| esr1.L | 12.13 | Estrogen |
| ABCC2 | 50 | ATP binding |
| - | 0.57 | - |
| - | 2.98 | - |
| - | 10 | - |
| - | 25.57 | - |
| CYP1A2 | 19.03 | Cytochrome |
| COL3A1 | 0.1 | Collagen |
| - | 0.21 | - |
| SULT2A1 | 50 | sulfotransferase |
| - | 9.14 | - |
| ABCG2 | 20.59 | ATP binding |
| - | 7.79 | - |
| - | 10 | - |
| - | 0.27 | - |
| ABCB1 | 25.47 | ATP binding |
| - | 14.71 | - |
| NR1I2 | 2.76 | Nuclear receptor |
| PGR | 66.46 | progesterone receptor |
| - | 40 | - |
| CYP2B6 | 7.55 | Cytochrome |
| - | 0.45 | - |
| pparg | 19.84 | Nuclear receptor |
| NR1I2 | 1.73 | Nuclear receptor |
| Pparg | 8.81 | Nuclear receptor |
| - | 10 | - |
| - | 0.05 | - |
| NFE2L2 | 50 | Nuclear receptor |
| - | 10 | - |
| - | 29.49 | - |
| NR1I2 | 0.42 | Nuclear receptor |
| AR | 23.65 | Androgen receptor |
| PPARG | 9.81 | Nuclear receptor |
| EGF | 50 | epidermal growth factor |
| - | 27.93 | - |
| - | 7.54 | - |
| SLC6A3 | 2.15 | Membrane receptor - liver |
| CYP2C19 | 13.59 | Cytochrome |
| ESR1 | 16.75 | Estrogen |
| CYP2B6 | 16.08 | Cytochrome |
| - | 7.7 | - |
| THRA \| THRB | 39.9 | thyroid hormone receptor |
| - | 27.94 | - |
| THBD | 32.19 | Membrane receptor - Thrombosis |
| - | 3.51 | - |
| PPARG | 19.84 | Nuclear receptor |
| - | 4.54 | - |
| UGT1A1 | 10 | glucuronidation |
| - | 1.72 | - |
| ESR1 | 15.16 | Estrogen |
| - | 46.17 | - |
| Tspo | 11.83 | Androgen receptor |
| - | 7.23 | - |
| esr2b | 32.56 | Estrogen |
| PPARG | 20.7 | Nuclear receptor |
| CYP2B6 | 20 | Cytochrome |
| CXCL10 | 12.65 | Inflammation |
| - | 5.88 | - |
| CYP2C9 | 12.91 | Cytochrome |
| - | 15 | - |
| CYP3A4 | 9.94 | Cytochrome |
| BACE1 | 3.66 | Neuroreceptor |
| - | 1.07 | - |
| PGR | 12.01 | progesterone receptor |
| CYP2C19 | 19.6 | Cytochrome |
| - | 1.15 | - |
| - | 3.15 | - |
| NR1H4 | 23.96 | Nuclear receptor |
| PPARG | 14.31 | Nuclear receptor |
| - | 44.2 | - |
| - | 70.98 | - |
| POU2F1 | 2.12 | DNA |
| HSPA1A | 17.23 | heat shock protein |
| - | 0.17 | - |
| CCL2 | 6.61 | Immune system receptor |
| - | 2.28 | - |
| - | 0.32 | - |
| CCL2 | 6.77 | Immune system receptor |
| - | 35.91 | - |
| CYP3A4 | 7.96 | Cytochrome |
| - | 32.78 | - |
| - | 1.66 | - |
| - | 15 | - |
| VDR | 7.39 | Nuclear receptor |
| NR1H2 \| NR1H3 | 14.27 | Nuclear receptor |
| - | 0.23 | - |
| NR1I2 | 21.99 | Nuclear receptor |
| - | 40 | - |
| PPARA \| PPARD \| PPARG | 7.71 | Nuclear receptor |
| Pparg | 8.82 | Nuclear receptor |
| CYP1A1 | 20 | Cytochrome |
| CYP2C8 | 15.5 | Cytochrome |
| - | 0.39 | - |
| esr2.L | 30.07 | Estrogen |
| - | 0.1 | - |
| NR3C1 | 6.93 | Nuclear receptor |
| RARA \| RARB \| RARG | 2.41 | Nuclear receptor |
| CYP1A1 | 45.28 | Cytochrome |
| - | 0.62 | - |
| NFE2L2 | 3.23 | Nuclear receptor |

**Table S5:** **Cypermethrin´s ToxCast information on the tissue categories**. Extracted information of Cypermethrin on its tissue and the corresponding AC50s extracted from the ToxCast database. The simplified tissue categories correspond to the categories given by the author for the purposes of this study.

| *TOXCAST BIOLOGICAL PROCESS TARGET* | | *TOXCAST TISSUE* | *TOXCAST AC50 VALUE (uM)* | | | *Simplified TISSUE CATEGORY* |
| --- | --- | --- | --- | --- | --- | --- |
| Transcription Factor Activity | liver | | | 18.73 | Liver | |
| Transcription Factor Activity | kidney | | | 24.17 | Kidney | |
| Transcription Factor Activity | liver | | | 10.74 | Liver | |
| Morphogenesis | ovary | | | 1.55 | Ovary | |
| Functional Neural Network Activity | cortical | | | 7.14 | Brain | |
| Receptor Binding | brain | | | 10.79 | Brain | |
| Transcription Factor Activity | liver | | | 11.96 | Liver | |
| Cell Proliferation | prostate | | | 70.87 | Prostate function | |
| Functional Neural Network Activity | cortical | | | 3.97 | Brain | |
| - | liver | | | 4.14 | - | |
| Transcription Factor Activity | kidney | | | 83.23 | Kidney | |
| - | liver | | | 2.01 | - | |
| Morphogenesis | ovary | | | 1.75 | Ovary | |
| Catalytic Activity | NA | | | 12.99 | - | |
| Functional Neural Network Activity | cortical | | | 1.94 | Brain | |
| Transcription Factor Activity | liver | | | 48.96 | Liver | |
| Morphogenesis | ovary | | | 0.93 | Ovary | |
| Dna Repair | lymphoblast | | | 66.95 | Immune system | |
| Gene Expression | vascular | | | 4.42 | Vascular system | |
| Functional Neural Network Activity | cortical | | | 0.62 | Brain | |
| Transcription Factor Activity | liver | | | 50 | Liver | |
| - | liver | | | 6.75 | - | |
| Spontaneous Neural Activity | cortical | | | 9.13 | Brain | |
| Transcription Factor Activity | liver | | | 100 | Liver | |
| Morphogenesis | ovary | | | 1.56 | Ovary | |
| Morphogenesis | ovary | | | 1.16 | Ovary | |
| Estrogen Receptor Activity | mammary gland/breast | | | 16.4 | Breast | |
| Transcription Factor Activity | liver | | | 25 | Liver | |
| Transcription Factor Activity | liver | | | 4.95 | Liver | |
| Transcription Factor Activity | liver | | | 27.59 | Liver | |
| Functional Neural Network Activity | cortical | | | 2.98 | Brain | |
| Transcription Factor Activity | liver | | | 66.48 | Liver | |
| - | liver | | | 10.99 | - | |
| Spontaneous Neural Activity | cortical | | | 1.57 | Brain | |
| Functional Neural Network Activity | cortical | | | 11.4 | Brain | |
| Transcription Factor Activity | liver | | | 10.67 | Liver | |
| Transcription Factor Activity | liver | | | 9.01 | Liver | |
| Catalytic Activity | NA | | | 4.96 |  | |
| Cell Proliferation | liver | | | 30.91 | Liver | |
| Transcription Factor Activity | liver | | | 50 | Liver | |
| Transcription Factor Activity | liver | | | 66.75 | Liver | |
| Transcription Factor Activity | liver | | | 2.41 | Liver | |
| Functional Neural Network Activity | cortical | | | 1.43 | Brain | |
| Transcription Factor Activity | liver | | | 11.01 | Liver | |
| - | liver | | | 6.46 | - | |
| Transcription Factor Activity | liver | | | 29.9 | Liver | |
| Transcription Factor Activity | liver | | | 6.45 | Liver | |
| Transcription Factor Activity | liver | | | 2.11 | Liver | |
| Receptor Binding | brain | | | 25 | Brain | |
| Receptor Binding | NA | | | 12.99 |  | |
| Transcription Factor Activity | liver | | | 15.52 | Liver | |
| Estrogen Receptor Activity | mammary gland/breast | | | 62.9 | Breast | |
| Cell Proliferation | vascular | | | 26.7 | Vascular system | |
| Transcription Factor Activity | liver | | | 12.52 | Liver | |
| Catalytic Activity | NA | | | 3.45 |  | |
| Cell Proliferation | prostate | | | 31.68 | Prostate function | |
| Cell Cycle | liver | | | 31.84 | Liver | |
| Spontaneous Neural Activity | cortical | | | 11.57 | Brain | |
| Catalytic Activity | NA | | | 6.6 |  | |
| Transcription Factor Activity | liver | | | 0.57 | Liver | |
| Transcription Factor Activity | liver | | | 50 | Liver | |
| Functional Neural Network Activity | cortical | | | 8.41 | Brain | |
| - | liver | | | 8.15 | - | |
| Morphogenesis | ovary | | | 1.63 | Ovary | |
| Receptor Binding | NA | | | 5.06 |  | |
| Spontaneous Neural Activity | cortical | | | - | - | |
| Transcription Factor Activity | pituitary gland | | | 47.33 | Brain | |
| Receptor Binding | pancreas | | | 20.44 | Pancreas | |
| Catalytic Activity | NA | | | 15.79 |  | |
| Transcription Factor Activity | liver | | | 16.67 | Liver | |
| Cell Death | vascular | | | 3.97 | Vascular system | |
| Functional Neural Network Activity | cortical | | | 1.37 | Brain | |
| Transcription Factor Activity | liver | | | 72.12 | Liver | |
| Transcription Factor Activity | liver | | | 13.04 | Liver | |
| Spontaneous Neural Activity | cortical | | | 2.98 | Brain | |
| - | liver | | | 1.54 | - | |
| Cell Death | vascular | | | 6.21 | Vascular system | |
| Transcription Factor Activity | liver | | | 16.39 | Liver | |
| Gene Expression | vascular | | | 7.08 | Vascular system | |
| Transcription Factor Activity | liver | | | 24.62 | Liver | |
| Functional Neural Network Activity | cortical | | | 3.6 | Brain | |
| Development | ovary | | | 0.26 | Ovary | |
| Receptor Binding | brain | | | 9.64 | Brain | |
| - | liver | | | 4.4 | - | |
| - | liver | | | 8.73 | - | |
| Transcription Factor Activity | liver | | | 13.75 | Liver | |
| Mitochondrial Depolarization | liver | | | 58.16 | Liver | |
| Cell Proliferation | liver | | | 73.1 | Liver | |
| - | liver | | | 2.15 | - | |
| Transcription Factor Activity | liver | | | 5.57 | Liver | |
| Transcription Factor Activity | liver | | | 18.14 | Liver | |
| Transcription Factor Activity | liver | | | 35.82 | Liver | |
| Receptor Binding | intestinal | | | 19.46 | Intestine | |
| Transcription Factor Activity | kidney | | | 21.24 | Kidney | |
| Estrogen Receptor Activity | mammary gland/breast | | | 68.15 | Breast | |
| Spontaneous Neural Activity | cortical | | | 6.35 | Brain | |
| Transcription Factor Activity | liver | | | 1.08 | Liver | |
| Morphogenesis | ovary | | | 1.71 | Ovary | |
| Gene Expression | vascular | | | 0.52 | Vascular system | |
| Morphogenesis | ovary | | | 1.44 | Ovary | |
| Spontaneous Neural Activity | cortical | | | 10.02 | Brain | |
| Transcription Factor Activity | cervix | | | 74.18 | Ovary | |
| Transcription Factor Activity | liver | | | 21.69 | Liver | |
| Estrogen Receptor Activity | mammary gland/breast | | | 56.64 | Breast | |
| Transcription Factor Activity | liver | | | 50 | Liver | |
| Transcription Factor Activity | liver | | | 3.58 | Liver | |
| Transcription Factor Activity | liver | | | 24.45 | Liver | |
| Catalytic Activity | NA | | | 14.16 |  | |
| Transcription Factor Activity | cervix | | | 32.22 | Ovary | |
| Cell Proliferation | vascular | | | 9.52 | Vascular system | |
| Cell Death | vascular | | | 6.03 | Vascular system | |
| Transcription Factor Activity | liver | | | 15.78 | Liver | |
| Gene Expression | vascular | | | 10.43 | Vascular system | |
| Transcription Factor Activity | liver | | | 28.35 | Liver | |
| - | liver | | | 3.01 | - | |
| - | liver | | | 14.61 | - | |
| Transcription Factor Activity | liver | | | 1.28 | Liver | |
| Catalytic Activity | NA | | | 10 |  | |
| Transcription Factor Activity | liver | | | 3.97 | Liver | |
| Spontaneous Neural Activity | cortical | | | 0.83 | Brain | |
| Receptor Binding | NA | | | 6.91 |  | |
| Morphogenesis | ovary | | | 1.07 | Ovary | |
| Transcription Factor Activity | liver | | | 9.34 | Liver | |
| Transcription Factor Activity | liver | | | 13.23 | Liver | |
| Receptor Binding | kidney | | | 5.61 | Kidney | |
| Transcription Factor Activity | liver | | | 11.38 | Liver | |
| Functional Neural Network Activity | cortical | | | 6.97 | Brain | |
| Spontaneous Neural Activity | cortical | | | 3.78 | Brain | |
| - | liver | | | 6.68 | - | |
| Cell Death | vascular | | | 9.37 | Vascular system | |
| Transcription Factor Activity | liver | | | 50 | Liver | |

**Table S6:** **Cypermethrin´s ToxCast information on the gene categories**. Extracted information of Cypermethrin on its gene activation and the corresponding AC50s extracted from the ToxCast database. The simplified gene category corresponds to the gene categories given by the author for the purposes of this study.

| TOXCAST GENE SYMBOL | *TOXCAST AC50 VALUE (uM)* | *SIMPLIFIED GENE CATEGORY* |
| --- | --- | --- |
| Pparg | 18.73 | Nuclear receptor |
| NR1H4 | 24.17 | Nuclear receptor |
| ESR1 | 10.74 | Estrogen |
| - | 1.55 | - |
| - | 7.14 | - |
| Cacna1a | 10.79 | Neuroreceptor |
| MTF1 | 11.96 | DNA |
| - | 70.87 | - |
| - | 3.97 | - |
| CYP3A4 | 4.14 | Cytochrome |
| ESRRA | 83.23 | Estrogen |
| CYP3A4 | 2.01 | Cytochrome |
| - | 1.75 | - |
| CYP2C9 | 12.99 | Cytochrome |
| - | 1.94 | - |
| CYP2E1 | 48.96 | Cytochrome |
| - | 0.93 | - |
| RAD54L \| XRCC6P1 | 66.95 | DNA |
| PTGER2 | 4.42 | Prostaglandin |
| - | 0.62 | - |
| PDK4 | 50 | Protein kinase |
| UGT1A1 | 6.75 | glucuronidation |
| - | 9.13 | - |
| esr2b | 100 | Estrogen |
| - | 1.56 | - |
| - | 1.16 | - |
| - | 16.4 | - |
| NR1I2 | 25 | Nuclear receptor |
| VDR | 4.95 | Nuclear receptor |
| NR1H2 \| NR1H3 | 27.59 | Nuclear receptor |
| - | 2.98 | - |
| ESR2 | 66.48 | Estrogen |
| CYP1A2 | 10.99 | Cytochrome |
| - | 1.57 | - |
| - | 11.4 | - |
| ABCG2 | 10.67 | ATP binding |
| NR1I2 | 9.01 | Nuclear receptor |
| MMP2 | 4.96 | Proteolysis |
| - | 30.91 | - |
| ABCB1 | 50 | ATP binding |
| esr2.L | 66.75 | Estrogen |
| NR1I2 | 2.41 | Nuclear receptor |
| - | 1.43 | - |
| pparg | 11.01 | Nuclear receptor |
| CYP2B6 | 6.46 | Cytochrome |
| esr1.L | 29.9 | Estrogen |
| PPARA \| PPARD \| PPARG | 6.45 | Nuclear receptor |
| NR1I2 | 2.11 | Nuclear receptor |
| CHRNA2 | 25 | Neuroreceptor |
| SLC6A2 | 12.99 | Neuroreceptor |
| ESR1 | 15.52 | Estrogen |
| ESR1 | 62.9 | Estrogen |
| - | 26.7 | - |
| Pparg | 12.52 | Nuclear receptor |
| DUSP3 | 3.45 | Protein phosphatase |
| AR | 31.68 | Androgen receptor |
| - | 31.84 | - |
| - | 11.57 | - |
| CYP2D6 | 6.6 | Cytochrome |
| NR1I2 | 0.57 | Nuclear receptor |
| EGF | 50 | epidermal growth factor |
| - | 8.41 | - |
| CYP1A1 | 8.15 | Cytochrome |
| - | 1.63 | - |
| HTR7 | 5.06 | Neuroreceptor |
| - | - | - |
| THRA \| THRB | 47.33 | thyroid hormone receptor |
| Cckar | 20.44 | Neuroreceptor |
| MMP9 | 15.79 | Proteolysis |
| UGT1A1 | 16.67 | glucuronidation |
| - | 3.97 | - |
| - | 1.37 | - |
| esr2b | 72.12 | Estrogen |
| PPARG | 13.04 | Nuclear receptor |
| - | 2.98 | - |
| CYP2B6 | 1.54 | Cytochrome |
| - | 6.21 | - |
| PPARG | 16.39 | Nuclear receptor |
| CCL2 | 7.08 | Immune system receptor |
| CYP2C19 | 24.62 | Cytochrome |
| - | 3.6 | - |
| - | 0.26 | - |
| Slc6a3 | 9.64 | Neuroreceptor |
| CYP2B6 | 4.4 | Cytochrome |
| CYP3A4 | 8.73 | Cytochrome |
| CYP7A1 | 13.75 | Cytochrome |
| - | 58.16 | - |
| - | 73.1 | - |
| CYP1A2 | 2.15 | Cytochrome |
| RARA \| RARB \| RARG | 5.57 | Nuclear receptor |
| ESR1 | 18.14 | Estrogen |
| CYP1A1 | 35.82 | Cytochrome |
| TSPO | 19.46 | Androgen receptor |
| PGR | 21.24 | progesterone receptor |
| ESR1 | 68.15 | Estrogen |
| - | 6.35 | - |
| NFE2L2 | 1.08 | Nuclear receptor |
| - | 1.71 | - |
| TNF | 0.52 | Proliferation |
| - | 1.44 | - |
| - | 10.02 | - |
| - | 74.18 | - |
| ESR1 | 21.69 | Estrogen |
| ESR1 | 56.64 | Estrogen |
| PEG10 | 50 | Apoptosis |
| pparg | 3.58 | Nuclear receptor |
| CYP1A2 | 24.45 | Cytochrome |
| MMP13 | 14.16 | Proteolysis |
| - | 32.22 | - |
| - | 9.52 | - |
| - | 6.03 | - |
| ESR1 | 15.78 | Estrogen |
| PLAUR | 10.43 | Plasminogen |
| NR1I3 | 28.35 | Nuclear receptor |
| CYP1A1 | 3.01 | Cytochrome |
| CYP1A2 | 14.61 | Cytochrome |
| ESR1 | 1.28 | Estrogen |
| CYP2C19 | 10 | Cytochrome |
| PPARG | 3.97 | Nuclear receptor |
| - | 0.83 | - |
| SLC6A3 | 6.91 | Neuroreceptor |
| - | 1.07 | - |
| CYP2B6 | 9.34 | Cytochrome |
| esr2.L | 13.23 | Estrogen |
| Tspo | 5.61 | Androgen receptor |
| CYP3A4 | 11.38 | Cytochrome |
| - | 6.97 | - |
| - | 3.78 | - |
| UGT1A1 | 6.68 | glucuronidation |
| - | 9.37 | - |
| IGF1 | 50 | Insulin |

**Table S7:** **Deltamethrin´s ToxCast information on the tissue categories**. Extracted information of Deltamethrin on its tissue and the corresponding AC50s extracted from the ToxCast database. The simplified tissue categories correspond to the categories given by the author for the purposes of this study.

| *TOXCAST BIOLOGICAL PROCESS TARGET* | | *TOXCAST TISSUE* | *TOXCAST AC50 VALUE (uM)* | | | *Simplified TISSUE CATEGORY* |
| --- | --- | --- | --- | --- | --- | --- |
| Transcription Factor Activity | kidney | | | 27.72 | Kidney | |
| Functional Neural Network Activity | cortical | | | 6.4 | Brain | |
| Cell Proliferation | breast | | | 69.69 | Breast | |
| Cell Proliferation | prostate | | | 50 | Prostate function | |
| Functional Neural Network Activity | cortical | | | 0.87 | Brain | |
| Spontaneous Neural Activity | cortical | | | 1.62 | Brain | |
| Spontaneous Neural Activity | cortical | | | 7.16 | Brain | |
| Transcription Factor Activity | kidney | | | 50 | Kidney | |
| Transcription Factor Activity | cervix | | | 71.07 | Ovary | |
| Functional Neural Network Activity | cortical | | | 0.64 | Brain | |
| Gene Expression | vascular | | | 2.14 | Vascular system | |
| Dna Repair | lymphoblast | | | 39.45 | Immune system | |
| Cell Proliferation | lymphoblast | | | 82.3 | Immune system | |
| Spontaneous Neural Activity | cortical | | | 5.29 | Brain | |
| Neurodevelopment | cortical | | | 28.38 | Brain | |
| Functional Neural Network Activity | cortical | | | 0.85 | Brain | |
| Gene Expression | vascular | | | 20 | Vascular system | |
| Spontaneous Neural Activity | cortical | | | 1.1 | Brain | |
| Transcription Factor Activity | liver | | | 7.36 | Liver | |
| Functional Neural Network Activity | cortical | | | 7.55 | Brain | |
| Cytotoxicity | brain | | | 1.05 | Brain | |
| Neurodevelopment | cortical | | | 8.81 | Brain | |
| Gene Expression | skin | | | 4.86 | Skin | |
| Cell Viability | brain | | | 0.44 | Brain | |
| Transcription Factor Activity | cervix | | | 20.31 | Ovary | |
| Transcription Factor Activity | kidney | | | 13.21 | Kidney | |
| Gene Expression | vascular | | | 8.7 | Vascular system | |
| Cell Proliferation | liver | | | 30.82 | Liver | |
| Functional Neural Network Activity | cortical | | | 0.64 | Brain | |
| Gene Expression | vascular | | | 0.15 | Vascular system | |
| Gene Expression | lung | | | 0.96 | Energy production - lung | |
| Spontaneous Neural Activity | cortical | | | 15 | Brain | |
| Gene Expression | vascular | | | 12.16 | Vascular system | |
| Functional Neural Network Activity | cortical | | | 1.5 | Brain | |
| Gene Expression | skin | | | 0.32 | Skin | |
| Spontaneous Neural Activity | cortical | | | 1.62 | Brain | |
| Spontaneous Neural Activity | cortical | | | 10.83 | Brain | |
| Cell Proliferation | vascular | | | 3.97 | Vascular system | |
| Transcription Factor Activity | liver | | | 38.67 | Liver | |
| Cell Proliferation | prostate | | | 50 | Prostate function | |
| Transcription Factor Activity | kidney | | | 74.88 | Kidney | |
| Gene Expression | vascular | | | 10.4 | Vascular system | |
| Catalytic Activity | NA | | | 2.05 | - | |
| Spontaneous Neural Activity | cortical | | | 2.1 | Brain | |
| Gene Expression | vascular | | | 1.74 | Vascular system | |
| Cell Proliferation | prostate | | | 30.97 | Prostate function | |
| Functional Neural Network Activity | cortical | | | 1.8 | Brain | |
| Gene Expression | vascular | | | 6.37 | Vascular system | |
| Transcription Factor Activity | liver | | | 0.59 | Liver | |
| Catalytic Activity | NA | | | 0.13 | - | |
| Transcription Factor Activity | intestinal | | | 86.41 | Intestine | |
| Gene Expression | vascular | | | 20 | Vascular system | |
| Transcription Factor Activity | cervix | | | 51.39 | Ovary | |
| Transcription Factor Activity | pituitary gland | | | 74.77 | Brain | |
| Transcription Factor Activity | intestinal | | | 80.28 | Intestine | |
| Spontaneous Neural Activity | cortical | | | 3.1 | Brain | |
| Functional Neural Network Activity | cortical | | | 0.2 | Brain | |
| Transcription Factor Activity | cervix | | | 67.64 | Ovary | |
| Neurodevelopment | cortical | | | 7.82 | Brain | |
| Transcription Factor Activity | liver | | | 0.56 | Liver | |
| Transcription Factor Activity | liver | | | 2.89 | Liver | |
| Spontaneous Neural Activity | cortical | | | 2.72 | Brain | |
| Functional Neural Network Activity | cortical | | | 0.64 | Brain | |
| Cytotoxicity | peripheral Brain | | | 115.03 | Brain | |
| Transcription Factor Activity | intestinal | | | 77.64 | Intestine | |
| Receptor Binding | intestinal | | | 9.86 | Intestine | |
| Functional Neural Network Activity | cortical | | | 3.96 | Brain | |
| Receptor Binding | kidney | | | 10.95 | Kidney | |
| Gene Expression | vascular | | | 2.23 | Vascular system | |
| Functional Neural Network Activity | cortical | | | 3.1 | Brain | |
| Mitochondrial Depolarization | liver | | | 41.95 | Liver | |
| Gene Expression | vascular | | | 1.99 | Vascular system | |
| Migration During Neurodevelopment | brain | | | 0.61 | Brain | |
| Migration During Neurodevelopment | brain | | | 0.08 | Brain | |
| Dna Repair | lymphoblast | | | 59.4 | Immune system | |
| Transcription Factor Activity | kidney | | | 0.46 | Kidney | |
| Spontaneous Neural Activity | cortical | | | 3.86 | Brain | |
| Transcription Factor Activity | kidney | | | 15.21 | Kidney | |
| Neurodevelopment | peripheral Brain | | | 111.02 | Brain | |
| Cell Proliferation | liver | | | 47.25 | Liver | |
| Gene Expression | vascular | | | 20 | Vascular system | |
| Gene Expression | vascular | | | 22.84 | Vascular system | |
| Gene Expression | vascular | | | 3.73 | Vascular system | |
| Transcription Factor Activity | cervix | | | 40 | Ovary | |
| Gene Expression | vascular | | | 0.12 | Vascular system | |
| Spontaneous Neural Activity | cortical | | | 0.38 | Brain | |
| Gene Expression | vascular | | | 3.97 | Vascular system | |
| Spontaneous Neural Activity | cortical | | | 0.39 | Brain | |
| Transcription Factor Activity | liver | | | 0.69 | Liver | |
| Transcription Factor Activity | liver | | | 0.17 | Liver | |
| Transcription Factor Activity | intestinal | | | 44.89 | Intestine | |
| Transcription Factor Activity | liver | | | 0.33 | Liver | |
| Gene Expression | vascular | | | 8.75 | Vascular system | |
| Receptor Binding | NA | | | 28.56 | - | |
| Receptor Binding | NA | | | 6.77 | - | |
| Neurodevelopment | cortical | | | 8.14 | Brain | |
| Neurodevelopment | brain | | | 0.71 | Brain | |
| Transcription Factor Activity | cervix | | | 56.55 | Ovary | |
| Functional Neural Network Activity | cortical | | | 3.82 | Brain | |
| Transcription Factor Activity | cervix | | | 60.88 | Ovary | |
| Functional Neural Network Activity | cortical | | | 2.04 | Brain | |
| Spontaneous Neural Activity | cortical | | | 2.53 | Brain | |
| Gene Expression | vascular | | | 0.28 | Vascular system | |
| Cell Proliferation | vascular | | | 20 | Vascular system | |
| Spontaneous Neural Activity | cortical | | | 3.71 | Brain | |

**Table S8:** **Deltamethrin´s ToxCast information on the gene categories**. Extracted information of Deltamethrin on its gene activation and the corresponding AC50s extracted from the ToxCast database. The simplified gene category corresponds to the gene categories given by the author for the purposes of this study.

| TOXCAST GENE SYMBOL | *TOXCAST AC50 VALUE (uM)* | *SIMPLIFIED GENE CATEGORY* |
| --- | --- | --- |
| NR1H4 | 27.72 | Nuclear receptor |
| - | 6.4 | - |
| - | 69.69 | - |
| - | 50 | - |
| - | 0.87 | - |
| - | 1.62 | - |
| - | 7.16 | - |
| ESRRA | 50 | Estrogen |
| - | 71.07 | - |
| - | 0.64 | - |
| PTGER2 | 2.14 | Prostaglandin |
| RAD54L \| XRCC6P1 | 39.45 | DNA |
| - | 82.3 | - |
| - | 5.29 | - |
| - | 28.38 | - |
| - | 0.85 | - |
| IL2 | 20 | Inflammation |
| - | 1.1 | - |
| NR1I2 | 7.36 | Nuclear receptor |
| - | 7.55 | - |
| - | 1.05 | - |
| - | 8.81 | - |
| COL3A1 | 4.86 | Collagen |
| - | 0.44 | - |
| - | 20.31 | - |
| - | 13.21 | - |
| PLAUR | 8.7 | Plasminogen |
| - | 30.82 | - |
| - | 0.64 | - |
| PLAUR | 0.15 | Plasminogen |
| HLA-DRA | 0.96 | Immune system receptor |
| - | 15 | - |
| IL6 | 12.16 | Inflammation |
| - | 1.5 | - |
| TIMP2 | 0.32 | Apoptosis |
| - | 1.62 | - |
| - | 10.83 | - |
| - | 3.97 | - |
| NR1I3 | 38.67 | Nuclear receptor |
| AR | 50 | Androgen receptor |
| - | 74.88 | - |
| IL17A | 10.4 | Inflammation |
| CYP2D6 | 2.05 | Cytochrome |
| - | 2.1 | - |
| SELE | 1.74 | Inflammation |
| - | 30.97 | - |
| - | 1.8 | - |
| THBD | 6.37 | Membrane receptor - Thrombosis |
| NR1I3 | 0.59 | Nuclear receptor |
| CYP2C19 | 0.13 | Cytochrome |
| - | 86.41 | - |
| IL17F | 20 | Inflammation |
| - | 51.39 | - |
| THRA \| THRB | 74.77 | thyroid hormone receptor |
| TP53 | 80.28 | Proliferation |
| - | 3.1 | - |
| - | 0.2 | - |
| HSF1 | 67.64 | heat shock protein |
| - | 7.82 | - |
| RARA \| RARB \| RARG | 0.56 | Nuclear receptor |
| PPARG | 2.89 | Nuclear receptor |
| - | 2.72 | - |
| - | 0.64 | - |
| - | 115.03 | - |
| - | 77.64 | - |
| TSPO | 9.86 | Androgen receptor |
| - | 3.96 | - |
| Tspo | 10.95 | Androgen receptor |
| CD69 | 2.23 | Proliferation |
| - | 3.1 | - |
| - | 41.95 | - |
| CD38 | 1.99 | Membrane receptor - Leukemia |
| - | 0.61 | - |
| - | 0.08 | - |
| REV3L | 59.4 | DNA |
| - | 0.46 | - |
| - | 3.86 | - |
| PGR | 15.21 | progesterone receptor |
| - | 111.02 | - |
| - | 47.25 | - |
| IL1A | 20 | Inflammation |
| IGHG1 | 22.84 | Immune system receptor |
| TNF | 3.73 | Proliferation |
| - | 40 | - |
| CCL26 | 0.12 | Immune system receptor |
| - | 0.38 | - |
| VCAM1 | 3.97 | Membrane receptor - artherosclerosis |
| - | 0.39 | - |
| VDR | 0.69 | Nuclear receptor |
| NR1I2 | 0.17 | Nuclear receptor |
| - | 44.89 | - |
| NR1I2 | 0.33 | Nuclear receptor |
| HLA-DRA | 8.75 | Immune system receptor |
| SLC6A2 | 28.56 | Neuroreceptor |
| SLC6A3 | 6.77 | Neuroreceptor |
| - | 8.14 | - |
| - | 0.71 | - |
| NR3C1 | 56.55 | Nuclear receptor |
| - | 3.82 | - |
| - | 60.88 | - |
| - | 2.04 | - |
| - | 2.53 | - |
| CCL2 | 0.28 | Immune system receptor |
| - | 20 | - |
| - | 3.71 | - |

**Table S9:** **L-Cyhalothrin´s ToxCast information on the tissue categories**. Extracted information of L-Cyhalothrin on its tissue and the corresponding AC50s extracted from the ToxCast database. The simplified tissue categories correspond to the categories given by the author for the purposes of this study.

| *TOXCAST BIOLOGICAL PROCESS TARGET* | | *TOXCAST TISSUE* | *TOXCAST AC50 VALUE (uM)* | | | *Simplified TISSUE CATEGORY* |
| --- | --- | --- | --- | --- | --- | --- |
| Functional Neural Network Activity | cortical | | | 15 | Brain | |
| Transcription Factor Activity | kidney | | | 24.4 | Kidney | |
| Cell Proliferation | skin | | | 6.94 | Skin | |
| Neurodevelopment | cortical | | | 27.75 | Brain | |
| Spontaneous Neural Activity | cortical | | | 1.35 | Brain | |
| Functional Neural Network Activity | cortical | | | 0.5 | Brain | |
| Cell Proliferation | breast | | | 40 | Breast | |
| Transcription Factor Activity | kidney | | | 44.97 | Kidney | |
| Transcription Factor Activity | cervix | | | 55.39 | Ovary | |
| Gene Expression | skin | | | 0.01 | Skin | |
| Functional Neural Network Activity | cortical | | | 0.59 | Brain | |
| Dna Repair | lymphoblast | | | 45.25 | Immune system | |
| Cell Proliferation | lymphoblast | | | 44.18 | Immune system | |
| Cell Proliferation | brain | | | 3.97 | Brain | |
| Functional Neural Network Activity | cortical | | | 0.95 | Brain | |
| Gene Expression | vascular | | | 0.04 | Vascular system | |
| Neurodevelopment | cortical | | | 2.66 | Brain | |
| Functional Neural Network Activity | cortical | | | 1.01 | Brain | |
| Neurodevelopment | cortical | | | 3.05 | Brain | |
| Transcription Factor Activity | liver | | | 3.48 | Liver | |
| Spontaneous Neural Activity | cortical | | | 1.01 | Brain | |
| Functional Neural Network Activity | cortical | | | 10.1 | Brain | |
| Gene Expression | vascular | | | 2.98 | Vascular system | |
| Transcription Factor Activity | liver | | | 0.14 | Liver | |
| Cell Death | cortical | | | 15 | Brain | |
| Neurodevelopment | cortical | | | 5.04 | Brain | |
| Neurodevelopment | cortical | | | 27.68 | Brain | |
| Functional Neural Network Activity | cortical | | | 1.55 | Brain | |
| Transcription Factor Activity | liver | | | 0.37 | Liver | |
| Neurodevelopment | cortical | | | 24.14 | Brain | |
| Gene Expression | lung | | | 0.01 | Energy production - lung | |
| Cell Proliferation | liver | | | 31.71 | Liver | |
| Spontaneous Neural Activity | cortical | | | 1.35 | Brain | |
| Gene Expression | skin | | | 0.35 | Skin | |
| Transcription Factor Activity | kidney | | | 35 | Kidney | |
| Gene Expression | vascular | | | 4.2 | Vascular system | |
| Cell Proliferation | vascular | | | 15 | Vascular system | |
| Cell Proliferation | kidney | | | 60.52 | Kidney | |
| Gene Expression | vascular | | | 15 | Vascular system | |
| Catalytic Activity | NA | | | 0.89 | - | |
| Spontaneous Neural Activity | cortical | | | 15 | Brain | |
| Gene Expression | vascular | | | 15 | Vascular system | |
| Spontaneous Neural Activity | cortical | | | 1.5 | Brain | |
| Transcription Factor Activity | pituitary gland | | | 35.75 | Brain | |
| Gene Expression | vascular | | | 4.6 | Vascular system | |
| Gene Expression | lung | | | 0.01 | Energy production - lung | |
| Neurodevelopment | cortical | | | 2.36 | Brain | |
| Functional Neural Network Activity | cortical | | | 0.04 | Brain | |
| Neurodevelopment | brain | | | 0.98 | Brain | |
| Catalytic Activity | NA | | | 6.32 | - | |
| Transcription Factor Activity | cervix | | | 51.13 | Ovary | |
| Functional Neural Network Activity | cortical | | | 4.01 | Brain | |
| Gene Expression | skin | | | 0.1 | Skin | |
| Cell Death | cortical | | | 28.15 | Brain | |
| Mitochondrial Depolarization | liver | | | 36.62 | Liver | |
| Functional Neural Network Activity | cortical | | | 0.47 | Brain | |
| Spontaneous Neural Activity | cortical | | | 1.4 | Brain | |
| Functional Neural Network Activity | cortical | | | 15 | Brain | |
| Gene Expression | vascular | | | 1.2 | Vascular system | |
| Neurodevelopment | cortical | | | 4.02 | Brain | |
| Neurodevelopment | cortical | | | 3.87 | Brain | |
| Neurodevelopment | cortical | | | 2.33 | Brain | |
| Transcription Factor Activity | liver | | | 6.8 | Liver | |
| Gene Expression | vascular | | | 2.98 | Vascular system | |
| Cell Proliferation | kidney | | | 28.21 | Kidney | |
| Spontaneous Neural Activity | cortical | | | 15 | Brain | |
| Gene Expression | vascular | | | 4.56 | Vascular system | |
| Gene Expression | vascular | | | 22.02 | Vascular system | |
| Catalytic Activity | NA | | | 0.93 | - | |
| Migration During Neurodevelopment | brain | | | 0.11 | Brain | |
| Cell Proliferation | kidney | | | 60.14 | Kidney | |
| Dna Repair | lymphoblast | | | 42.83 | Immune system | |
| Gene Expression | vascular | | | 18.94 | Vascular system | |
| Spontaneous Neural Activity | cortical | | | 27.89 | Brain | |
| Gene Expression | lung | | | 1.75 | Energy production - lung | |
| Gene Expression | vascular | | | 0.69 | Vascular system | |
| Spontaneous Neural Activity | cortical | | | 0.8 | Brain | |
| Spontaneous Neural Activity | cortical | | | 0.73 | Brain | |
| Gene Expression | vascular | | | 15 | Vascular system | |
| Neurodevelopment | cortical | | | 2.75 | Brain | |
| Transcription Factor Activity | kidney | | | 6.96 | Kidney | |
| Cell Viability | brain | | | 0.64 | Brain | |
| Functional Neural Network Activity | cortical | | | 0.59 | Brain | |
| Gene Expression | vascular | | | 15 | Vascular system | |
| Functional Neural Network Activity | cortical | | | 5.68 | Brain | |
| Transcription Factor Activity | kidney | | | 26.98 | Kidney | |
| Gene Expression | vascular | | | 0.01 | Vascular system | |
| Gene Expression | skin | | | 0.34 | Skin | |
| Gene Expression | vascular | | | 2.98 | Vascular system | |

**Table S10:** **L-Cyhalothrin´s ToxCast information on the gene categories**. Extracted information of L-Cyhalothrin on its gene activation and the corresponding AC50s extracted from the ToxCast database. The simplified gene category corresponds to the gene categories given by the author for the purposes of this study.

| TOXCAST GENE SYMBOL | *TOXCAST AC50 VALUE (uM)* | *SIMPLIFIED GENE CATEGORY* |
| --- | --- | --- |
| - | 15 | - |
| NR1H4 | 24.4 | Nuclear receptor |
| - | 6.94 | - |
| - | 27.75 | - |
| - | 1.35 | - |
| - | 0.5 | - |
| - | 40 | - |
| ESRRA | 44.97 | Estrogen |
| - | 55.39 | - |
| MMP1 | 0.01 | Proteolysis |
| - | 0.59 | - |
| RAD54L \| XRCC6P1 | 45.25 | DNA |
| - | 44.18 | - |
| - | 3.97 | - |
| - | 0.95 | - |
| CXCL8 | 0.04 | Inflammation |
| - | 2.66 | - |
| - | 1.01 | - |
| - | 3.05 | - |
| NR1I2 | 3.48 | Nuclear receptor |
| - | 1.01 | - |
| - | 10.1 | - |
| MMP1 | 2.98 | Proteolysis |
| NR1I2 | 0.14 | Nuclear receptor |
| - | 15 | - |
| - | 5.04 | - |
| - | 27.68 | - |
| - | 1.55 | - |
| NR1I2 | 0.37 | Nuclear receptor |
| - | 24.14 | - |
| HLA-DRA | 0.01 | Immune system receptor |
| - | 31.71 | - |
| - | 1.35 | - |
| TIMP2 | 0.35 | Apoptosis |
| - | 35 | - |
| CD40 | 4.2 | Immune system receptor |
| - | 15 | - |
| - | 60.52 | - |
| IL6 | 15 | Inflammation |
| CYP2C19 | 0.89 | Cytochrome |
| - | 15 | - |
| IL17A | 15 | Inflammation |
| - | 1.5 | - |
| THRA \| THRB | 35.75 | thyroid hormone receptor |
| THBD | 4.6 | Membrane receptor - Thrombosis |
| MMP1 | 0.01 | Proteolysis |
| - | 2.36 | - |
| - | 0.04 | - |
| - | 0.98 | - |
| MMP9 | 6.32 | Proteolysis |
| HSF1 | 51.13 | heat shock protein |
| - | 4.01 | - |
| PLAU | 0.1 | Plasminogen |
| - | 28.15 | - |
| - | 36.62 | - |
| - | 0.47 | - |
| - | 1.4 | - |
| - | 15 | - |
| CCL2 | 1.2 | Immune system receptor |
| - | 4.02 | - |
| - | 3.87 | - |
| - | 2.33 | - |
| PPARG | 6.8 | Nuclear receptor |
| LDLR | 2.98 | Fatty acid |
| - | 28.21 | - |
| - | 15 | - |
| CD38 | 4.56 | Membrane receptor - Leukemia |
| IGHG1 | 22.02 | Immune system receptor |
| CYP2C18 | 0.93 | Cytochrome |
| - | 0.11 | - |
| - | 60.14 | - |
| REV3L | 42.83 | DNA |
| IL1A | 18.94 | Inflammation |
| - | 27.89 | - |
| PLAU | 1.75 | Plasminogen |
| PTGER2 | 0.69 | Prostaglandin |
| - | 0.8 | - |
| - | 0.73 | - |
| IL2 | 15 | Inflammation |
| - | 2.75 | - |
| AR | 6.96 | Androgen receptor |
| - | 0.64 | - |
| - | 0.59 | - |
| IL17F | 15 | Inflammation |
| - | 5.68 | - |
| PGR | 26.98 | progesterone receptor |
| SERPINE1 | 0.01 | Plasminogen |
| MMP9 | 0.34 | Proteolysis |
| CD69 | 2.98 | Proliferation |

**Table S11:** **Permethrin´s ToxCast information on the tissue categories**. Extracted information of Permethrin on its tissue and the corresponding AC50s extracted from the ToxCast database. The simplified tissue categories correspond to the categories given by the author for the purposes of this study.

| *TOXCAST BIOLOGICAL PROCESS TARGET* | | *TOXCAST TISSUE* | *TOXCAST AC50 VALUE (uM)* | | | *Simplified TISSUE CATEGORY* |
| --- | --- | --- | --- | --- | --- | --- |
| Transcription Factor Activity | liver | | | 19.62 | Liver | |
| Functional Neural Network Activity | cortical | | | 10 | Brain | |
| Neurodevelopment | cortical | | | 92.65 | Brain | |
| Cell Proliferation | prostate | | | 27.73 | Prostate function | |
| Functional Neural Network Activity | cortical | | | 34.99 | Brain | |
| Cell Proliferation | skin | | | 5.91 | Skin | |
| Spontaneous Neural Activity | cortical | | | 7.5 | Brain | |
| Functional Neural Network Activity | cortical | | | 20 | Brain | |
| Transcription Factor Activity | liver | | | 3.74 | Liver | |
| Gene Expression | vascular | | | 5.19 | Vascular system | |
| - | liver | | | 3.97 | Liver | |
| Gene Expression | vascular | | | 6.16 | Vascular system | |
| Transcription Factor Activity | liver | | | 100 | Liver | |
| Gene Expression | skin | | | 6.34 | Skin | |
| Estrogen Receptor Activity | mammary gland/breast | | | 71.68 | Breast | |
| Transcription Factor Activity | liver | | | 10.89 | Liver | |
| Transcription Factor Activity | liver | | | 27.2 | Liver | |
| Neurodevelopment | cortical | | | 5.69 | Brain | |
| Neurodevelopment | cortical | | | 8.65 | Brain | |
| Functional Neural Network Activity | cortical | | | 9.02 | Brain | |
| Transcription Factor Activity | liver | | | 18.27 | Liver | |
| Cell Proliferation | vascular | | | 3.88 | Vascular system | |
| Spontaneous Neural Activity | cortical | | | 20 | Brain | |
| Cell Death | vascular | | | 4.15 | Vascular system | |
| Cell Death | cortical | | | 14.58 | Brain | |
| Gene Expression | vascular | | | 7.89 | Vascular system | |
| Cell Proliferation | kidney | | | 40 | Kidney | |
| Transcription Factor Activity | liver | | | 7.09 | Liver | |
| Spontaneous Neural Activity | cortical | | | 10 | Brain | |
| Gene Expression | vascular | | | 4.47 | Vascular system | |
| Neurodevelopment | cortical | | | 93.19 | Brain | |
| Neurodevelopment | cortical | | | 76.26 | Brain | |
| Transcription Factor Activity | liver | | | 10.91 | Liver | |
| - | liver | | | 9.51 | Liver | |
| Transcription Factor Activity | liver | | | 9.18 | Liver | |
| - | liver | | | 3.87 | Liver | |
| Transcription Factor Activity | liver | | | 1.51 | Liver | |
| Transcription Factor Activity | liver | | | 5.32 | Liver | |
| Cell Morphology | vascular | | | 12.27 | Vascular system | |
| Spontaneous Neural Activity | cortical | | | 28.13 | Brain | |
| Cell Proliferation | prostate | | | 29.71 | Prostate function | |
| Spontaneous Neural Activity | cortical | | | 10 | Brain | |
| Transcription Factor Activity | liver | | | 8.63 | Liver | |
| Protein Stabilization | liver | | | 100 | Liver | |
| Cell Proliferation | kidney | | | 40 | Kidney | |
| Steroid Hormone Biosynthetic Process | adrenal gland | | | 10 | Brain | |
| Cell Proliferation | vascular | | | 10.52 | Vascular system | |
| Gene Expression | vascular | | | 8.25 | Vascular system | |
| Cell Cycle | liver | | | 102.31 | Liver | |
| Transcription Factor Activity | liver | | | 18.01 | Liver | |
| Neurodevelopment | cortical | | | 8.69 | Brain | |
| Transcription Factor Activity | liver | | | 2.24 | Liver | |
| Cell Death | liver | | | 63.5 | Liver | |
| Cell Death | vascular | | | 4.38 | Vascular system | |
| Functional Neural Network Activity | cortical | | | 6.02 | Brain | |
| Transcription Factor Activity | liver | | | 14.42 | Liver | |
| Steroid Hormone Biosynthetic Process | adrenal gland | | | 7.84 | Brain | |
| Cell Death | cortical | | | 9.48 | Brain | |
| Transcription Factor Activity | kidney | | | 14.46 | Kidney | |
| Functional Neural Network Activity | cortical | | | 6.22 | Brain | |
| Steroid Hormone Biosynthetic Process | adrenal gland | | | 10.13 | Brain | |
| Transcription Factor Activity | liver | | | 38.55 | Liver | |
| Functional Neural Network Activity | cortical | | | 10 | Brain | |
| Neurodevelopment | cortical | | | 9.11 | Brain | |
| Development | ovary | | | 3.36 | Ovary | |
| Transcription Factor Activity | liver | | | 6.51 | Liver | |
| Catalytic Activity | NA | | | 46.82 | - | |
| Transcription Factor Activity | liver | | | 15.52 | Liver | |
| Estrogen Receptor Activity | mammary gland/breast | | | 80.36 | Breast | |
| Gene Expression | vascular | | | 20 | Vascular system | |
| - | liver | | | 7.12 | Liver | |
| Transcription Factor Activity | liver | | | 100 | Liver | |
| Transcription Factor Activity | liver | | | 25.58 | Liver | |
| Gene Expression | vascular | | | 3.87 | Vascular system | |
| Spontaneous Neural Activity | cortical | | | 27.85 | Brain | |
| Functional Neural Network Activity | cortical | | | 6 | Brain | |
| Neurodevelopment | cortical | | | 8.52 | Brain | |
| Transcription Factor Activity | liver | | | 9.41 | Liver | |
| Transcription Factor Activity | liver | | | 13.8 | Liver | |
| Gene Expression | vascular | | | 4.05 | Vascular system | |
| Gene Expression | skin | | | 5.08 | Skin | |
| Functional Neural Network Activity | cortical | | | 4.99 | Brain | |
| Neurodevelopment | cortical | | | 78.17 | Brain | |
| Steroid Hormone Biosynthetic Process | adrenal gland | | | 10.31 | Brain | |
| Transcription Factor Activity | liver | | | 7.43 | Liver | |
| Transcription Factor Activity | liver | | | 33.56 | Liver | |
| Transcription Factor Activity | liver | | | 2.62 | Liver | |
| Transcription Factor Activity | liver | | | 54.39 | Liver | |
| Transcription Factor Activity | liver | | | 40.61 | Liver | |
| Cell Proliferation | prostate | | | 30.79 | Prostate function | |
| Functional Neural Network Activity | cortical | | | 6.06 | Brain | |
| Transcription Factor Activity | liver | | | 11.82 | Liver | |
| Transcription Factor Activity | liver | | | 19.84 | Liver | |
| Transcription Factor Activity | liver | | | 27.86 | Liver | |
| Cell Death | vascular | | | 4.85 | Vascular system | |
| - | liver | | | 0.08 | Liver | |
| Mitochondrial Depolarization | liver | | | 100 | Liver | |
| Transcription Factor Activity | liver | | | 22.09 | Liver | |
| Receptor Binding | intestinal | | | 46.82 | Intestine | |
| Spontaneous Neural Activity | cortical | | | 27.58 | Brain | |
| Spontaneous Neural Activity | cortical | | | 10 | Brain | |
| Transcription Factor Activity | liver | | | 23.45 | Liver | |
| Cell Proliferation | kidney | | | 40 | Kidney | |

**Table S12:** **Permethrin´s ToxCast information on the gene categories**. Extracted information of Permethrin on its gene activation and the corresponding AC50s extracted from the ToxCast database. The simplified gene category corresponds to the gene categories given by the author for the purposes of this study.

| TOXCAST GENE SYMBOL | *TOXCAST AC50 VALUE (uM)* | *SIMPLIFIED GENE CATEGORY* |
| --- | --- | --- |
| ESR1 | 19.62 | Estrogen |
| - | 10 | - |
| - | 92.65 | - |
| - | 27.73 | - |
| - | 34.99 | - |
| - | 5.91 | - |
| - | 7.5 | - |
| - | 20 | - |
| ESR1 | 3.74 | Estrogen |
| IL6 | 5.19 | Inflammation |
| UGT1A1 | 3.97 | glucuronidation |
| CXCL8 | 6.16 | Inflammation |
| esr2b | 100 | Estrogen |
| EGFR | 6.34 | Protein kinase |
| ESR1 | 71.68 | Estrogen |
| CYP1A2 | 10.89 | Cytochrome |
| NR1H2 \| NR1H3 | 27.2 | Nuclear receptor |
| - | 5.69 | - |
| - | 8.65 | - |
| - | 9.02 | - |
| NR1I2 | 18.27 | Nuclear receptor |
| - | 3.88 | - |
| - | 20 | - |
| - | 4.15 | - |
| - | 14.58 | - |
| CXCL8 | 7.89 | Inflammation |
| - | 40 | - |
| ESR1 | 7.09 | Estrogen |
| - | 10 | - |
| HLA-DRA | 4.47 | Immune system receptor |
| - | 93.19 | - |
| - | 76.26 | - |
| PPARA \| PPARD \| PPARG | 10.91 | Nuclear receptor |
| CYP1A2 | 9.51 | Cytochrome |
| Pparg | 9.18 | Nuclear receptor |
| CYP2B6 | 3.87 | Cytochrome |
| NR1I2 | 1.51 | Nuclear receptor |
| NR1I3 | 5.32 | Nuclear receptor |
| - | 12.27 | - |
| - | 28.13 | - |
| AR | 29.71 | Androgen receptor |
| - | 10 | - |
| PPARG | 8.63 | Nuclear receptor |
| TUBA1A | 100 | Neuroreceptor |
| - | 40 | - |
| PGR | 10 | progesterone receptor |
| - | 10.52 | - |
| THBD | 8.25 | Membrane receptor - Thrombosis |
| - | 102.31 | - |
| CYP3A7 | 18.01 | Cytochrome |
| - | 8.69 | - |
| esr2.L | 2.24 | Estrogen |
| - | 63.5 | - |
| - | 4.38 | - |
| - | 6.02 | - |
| UGT1A1 | 14.42 | glucuronidation |
| PGR | 7.84 | progesterone receptor |
| - | 9.48 | - |
| PGR | 14.46 | progesterone receptor |
| - | 6.22 | - |
| NR3C1 \| NR3C2 | 10.13 | Nuclear receptor |
| CYP1A1 | 38.55 | Cytochrome |
| - | 10 | - |
| - | 9.11 | - |
| - | 3.36 | - |
| NFE2L2 | 6.51 | Nuclear receptor |
| BACE1 | 46.82 | Neuroreceptor |
| esr2a | 15.52 | Estrogen |
| ESR1 | 80.36 | Estrogen |
| CD69 | 20 | Proliferation |
| CYP2B6 | 7.12 | Cytochrome |
| THRB | 100 | thyroid hormone receptor |
| CYP2E1 | 25.58 | Cytochrome |
| PTGER2 | 3.87 | Prostaglandin |
| - | 27.85 | - |
| - | 6 | - |
| - | 8.52 | - |
| VDR | 9.41 | Nuclear receptor |
| NR1I2 | 13.8 | Nuclear receptor |
| CSF1 | 4.05 | Proteolysis |
| COL3A1 | 5.08 | Collagen |
| - | 4.99 | - |
| - | 78.17 | - |
| PGR | 10.31 | progesterone receptor |
| NR1I2 | 7.43 | Nuclear receptor |
| esr2.L | 33.56 | Estrogen |
| NR1I2 | 2.62 | Nuclear receptor |
| Pparg | 54.39 | Nuclear receptor |
| ESR1 | 40.61 | Estrogen |
| - | 30.79 | - |
| - | 6.06 | - |
| CYP2B6 | 11.82 | Cytochrome |
| esr2b | 19.84 | Estrogen |
| PPARG | 27.86 | Nuclear receptor |
| - | 4.85 | - |
| CYP2B6 | 0.08 | Cytochrome |
| - | 100 | - |
| CYP3A4 | 22.09 | Cytochrome |
| TSPO | 46.82 | Androgen receptor |
| - | 27.58 | - |
| - | 10 | - |
| ESR1 | 23.45 | Estrogen |
| - | 40 | - |

# Appendix 2:

- **Cypermethrin – Brain**: Cypermethrin exhibits activation in both the pituitary gland and brain, with higher AC_50_ values observed in these regions. Additionally, it is active in two related biological processes at the cortical level - functional neural network activity and spontaneous neural activity (extracellular) - with lower AC_50_ values for these endpoints. Significant variability was observed between the two neural activity endpoints, with greater bioactivity noted in neural activity than in neurodevelopment. An investigation was undertaken to determine whether other pyrethroids follow this same pattern but with different potencies, or if they involve distinct activities:
  - **Bifenthrin**: The most potent pyrethroid in the brain, also activates functional neural network activity and spontaneous neural activity, following the same pattern as Cypermethrin.
  - **Cyfluthrin and deltamethrin**: These compounds similarly activate functional neural network activity and spontaneous neural activity but also engage in a cellular neurodevelopment process.
  - **L-cyhalothrin and permethrin**: Both activate the three processes mentioned above (functional neural network activity, spontaneous neural activity, and neurodevelopment) and additionally induce cell death at the mitochondrial level.
- **Deltamethrin – Lung**: Deltamethrin disrupts the regulation of gene expression pathway in the lungs. The goal here has been to assess whether other substances, even those not flagged as highly active, also affect this pathway, potentially revealing a common pattern. Specifically, deltamethrin activates the MHC class II pathway with an AC_50_ of 0.96. The aim was to determine if other compounds followed a similar pattern but with varying toxicological potencies:
  - **L-cyhalothrin**: This compound also disrupts the regulation of gene expression pathway, but through three different proteins: MHC class II, matrix metalloproteinase, and serine protease. Its toxicological potency is much greater, with an AC_50_ of 0.01.
  - **Other Pyrethroids**: None of the other pyrethroids show activity in the lungs.
- **Deltamethrin – Skin**: Deltamethrin only affects gene expression regulation in the skin. Its behavior is similar to that of l-cyhalothrin, though with significantly different AC_50_ values and the involvement of different proteins (collagen and matrix metalloproteinase). Proliferation was measured and found to be inactive in deltamethrin, while it was active in l-cyhalothrin and permethrin.
- **L-cyhalothrin – Skin**: L-cyhalothrin activates two primary groups in the skin, cellular proliferation and gene expression regulation. Some variability was observed in the biological assay, particularly in the case of matrix metalloproteinase. Collagen III was measured and found to be inactive in l-cyhalothrin, although it was active in deltamethrin and permethrin. Dose-response curves were examined, suggesting the possibility of a non-monotonic response.
- **Permethrin – Skin**: The AC_50_ values for permethrin fall within a similar range to those of other compounds, though the slopes of the dose-response curves appear distinct. In the case of the tyrosine kinase receptor, the AC10 value is much higher than that of the other endpoints, indicating a much steeper dose-response relationship. In the skin, permethrin activates two groups: cellular proliferation and gene expression regulation. Three different effects were observed for these processes, despite having similar AC_50_ values (proliferation, collagen, and tyrosine kinase receptor activation). TIMP1 (a metalloproteinase inhibitor) was measured and found to be inactive, while TIMP2, which is active in deltamethrin and l-cyhalothrin, was not measured for permethrin.
